# Fluoropyrimidine Chemotherapy and the Risk of Death and Cardiovascular Events in Patients With Gastrointestinal Cancer

**DOI:** 10.1016/j.jaccao.2025.01.019

**Published:** 2025-04-08

**Authors:** Aderonke T. Abiodun, Chengsheng Ju, Catherine A. Welch, Jennifer Lai, Freya Tyrer, Pinkie Chambers, Lizz Paley, Sally Vernon, NICOR Collaborative, Andrew Goodwin, Peter Ludman, Theresa McDonagh, Clive Weston, John Deanfield, Mark de Belder, Mark J. Rutherford, Paul C. Lambert, Sarah Slater, Kai-Keen Shiu, Li Wei, Michael D. Peake, David Adlam, Charlotte Manisty

**Affiliations:** a Institute of Cardiovascular Science, University College London, United Kingdom; b Barts Heart Centre, Barts Health NHS Trust, London, United Kingdom; c National Disease Registration Service, NHS England, Wellington Place, Leeds, United Kingdom; d Research Department of Practice and Policy, School of Pharmacy, University College London, United Kingdom; e Department of Population Health Sciences, University of Leicester, Leicester, United Kingdom; f Cancer Division, University College London Hospitals NHS Foundation Trust, London, United Kingdom; g Centre for Medicines Optimization Research and Education, University College London Hospitals NHS Foundation Trust, London, United Kingdom; h National Institute of Cardiovascular Outcomes Research, NHS Arden and Greater East Midlands Commissioning Support Unit, Leicester, United Kingdom; i Department of Medical Epidemiology and Biostatistics, Karolinska Institute, Stockholm, Sweden; j Barts Cancer Centre, Barts Health NHS Trust, London, United Kingdom; k Cancer Institute, University College London, London, United Kingdom; l Department of Respiratory Medicine, University of Leicester, Glenfield Hospital, Leicester, United Kingdom; m Cancer Research UK, London, England; n Department of Cardiovascular Sciences and NIHR Leicester Biomedical Research Centre, University of Leicester, Glenfield Hospital, Leicester, United Kingdom

**Keywords:** 5-fluorouracil, arrhythmia, capecitabine, cardiotoxicity, gastrointestinal cancer, outcomes, risk prediction, sudden death, target trial emulation, vasospasm

## Abstract

**Background:**

Fluoropyrimidine chemotherapy is administered first-line for many gastrointestinal cancers. However, patients with cardiovascular disease commonly receive alternative treatment due to cardiotoxicity concerns.

**Objectives:**

This study sought to assess the risks of all-cause mortality and acute cardiovascular events with fluoropyrimidine treatment.

**Methods:**

We conducted an observational cohort study applying a target trial emulation framework to linked national cancer, cardiac, and hospitalization registry data from the Virtual Cardio-Oncology Research Initiative. Adults diagnosed with tumors eligible for fluoropyrimidine-based chemotherapy as first-line therapy were included. All-cause mortality and a composite of hospitalization for acute cardiovascular events (acute coronary syndrome, heart failure, cardiac arrhythmia, cardiac intervention, cardiac arrest, and cardiac death) were compared in patients treated with fluoropyrimidine-based chemotherapy vs alternative management. Adjusted, weighted pooled logistic regression models were used to estimate the 1-year risk difference (RD).

**Results:**

Among 103,110 patients (mean age 69.7 years, 59% male), the absolute risk of death at 1 year was significantly lower in fluoropyrimidine-treated patients (RD: −7.7%; 95% CI: −8.7% to −6.7%) with a small increased risk of acute cardiovascular events (RD: 0.9%; 95% CI: 0.0% to 1.9%). This was primarily due to arrhythmias (RD: 0.8%; 95% CI: 0.1% to 1.6%) and cardiac arrest (RD: 0.3%; 95% CI: 0.1% to 0.5%), with no increased risk of acute coronary syndromes including in the subgroup of patients with pre-existing coronary artery disease.

**Conclusions:**

The markedly improved overall survival with fluoropyrimidines in patients with gastrointestinal cancer significantly outweighs the small risk of cardiac arrhythmia and arrest. Oncologists should take this into consideration for decision making to avoid undue clinical conservatism, particularly in patients with cardiovascular disease.

Fluoropyrimidine chemotherapy agents are used first line in the treatment of many gastrointestinal and other solid organ malignancies globally.^1^, ^2^, ^3^ The development of cardiotoxicity can often complicate its use and has various manifestations from asymptomatic electrocardiogram changes to angina, heart failure (HF), myocardial infarction, and sudden cardiac death.^4^ Baseline cardiovascular disease (CVD) and cardiovascular risk factors are common in patients with gastrointestinal malignancies.^5^ Hence, many cancer clinicians exercise caution in prescribing fluoropyrimidines for patients with established CVD due to a perceived elevated risk of fluoropyrimidine-associated cardiotoxicity, although the incidence, severity, and mechanism of fluoropyrimidine-associated cardiotoxicity have been inconsistently reported in previous studies.^6^ Fluoropyrimidine-associated cardiotoxicity events most frequently occur during the first cycle of treatment and within days of treatment administration.^7^

To date, no clinical trial has compared the risk of cardiovascular events of fluoropyrimidine-based treatment vs no fluoropyrimidine treatment in a real-world clinical setting. A future randomized controlled trial (RCT) could generate such evidence; however, this may be ethically challenging, given established data supporting clinical efficacy and the lack of available alternatives.^8^ Therefore, using observational data to emulate the target trial may be the best alternative approach for estimating the effectiveness of fluoropyrimidine treatment^9^ in the cancer population.

Using national disease registries from England, we emulated a target trial to examine the risk of all-cause mortality and acute cardiovascular events in patients with eligible gastrointestinal cancer receiving fluoropyrimidine treatment vs no fluoropyrimidine treatment.

## Methods

### Data source

We used data provided by the Virtual Cardio-Oncology Research Initiative (VICORI) program for this study.^10^ The VICORI is a research platform that links the National Cancer Registration Dataset (NCRD), National Institute for Cardiovascular Outcomes Research data for CVD registries, Hospital Episode Statistics for hospital admission data, and Office for National Statistics death registry data through a unique identifier. The NCRD was also linked with the SACT (Systemic Anti-Cancer Therapy) database for the identification of systemic anticancer therapies for the patient population.^11^ The VICORI program has ethical approval from the Northeast–Newcastle and North Tyneside 2 Research Ethics Committee (reference 18/NE/0123).

### Study design and study population

We conducted an observational cohort study following the target trial emulation framework.^12^ First, we designed a pragmatic target trial that would answer the causal question of interest; second, we emulated this using data from VICORI. The specifications of the target trial and trial emulation are presented in Supplemental Table 1.

Patients 18 to 100 years of age in England with a first diagnosis of a gastrointestinal cancer (International Classification of Diseases–10th Revision [ICD-10] codes: C15, C16, C18-C21) between January 2014 and March 2018 were identified from the NCRD data. We only included patients with recorded stage II to IV esophageal or gastric cancers, or stage III or IV colorectal cancer, for which neoadjuvant/adjuvant or palliative fluoropyrimidine is guideline indicated.^13^^,^^14^ We excluded patients with tumor histology types for which fluoropyrimidines are not indicated (melanoma, sarcoma, carcinoid, neuroendocrine, mesenchymal, and Paget’s disease). We further excluded patients with missing data on vital status or no follow-up (ie, died on the same date as the cancer diagnosis) and cancer stage (noninferable) and patients with a cardiovascular event (acute coronary syndrome [ACS], HF and cardiomyopathy, coronary interventions, cardiac arrhythmia, and cardiac arrest) within 60 days prior to cancer diagnosis, to prevent capturing repeated recordings of the index event as an outcome event (as applied in previous trial emulations).^15^ All patients were followed for 1 year from cancer diagnosis until study outcome or death, whichever occurred first.

### Treatment strategies

We compared the treatment strategies of receiving a fluoropyrimidine (5-fluouracil or capecitabine) chemotherapy–based regimen to not receiving fluoropyrimidine chemotherapy in the included patients. Receipt of fluoropyrimidine treatment was defined as administration of a fluoropyrimidine treatment within 8 weeks (ie, a grace period) after the first gastrointestinal cancer diagnosis. In sensitivity analysis, we extended the grace period to 12 and 16 weeks. All patients could have received other cancer treatments where appropriate.

### Study outcomes

The study outcomes were 1-year all-cause mortality, and a 1-year composite of hospitalization for cardiovascular events (including ACS, HF and cardiomyopathy, coronary interventions, cardiac arrhythmia, and cardiac arrest and cardiovascular death). Cardiovascular events as study outcomes were restricted to the first 3 diagnostic codes in the hospital records for the admission.

### Covariates

The covariates included in the analysis included age at the cancer diagnosis, sex, ethnicity group (White, Asian, Black, mixed, and other), Charlson Comorbidity Index from 78 months to 6 months prior to the cancer diagnosis, Index of Multiple Deprivation quintiles for socioeconomic status, tumor site, tumor stage, tumor grade, route of cancer diagnosis, previous hospitalization within 10 years with the diagnosis of hypertension, type 2 diabetes mellitus, chronic obstructive pulmonary disease, chronic kidney disease, coronary artery disease, venous thromboembolism, HF and cardiomyopathy, valvular disease, cardiac arrhythmia, stroke, peripheral vascular disease, cardiac arrest, and pericarditis. All available positions of the diagnosis from any hospital admissions were used to capture the baseline covariates.

The definitions of all study variables are provided in Supplemental Table 2.

### Statistical analysis and emulation of the target trial

The target trial with the clone-censor-weight method has been recommended for investigating effectiveness and safety of cancer treatment strategies, which the treatment status (ie, the receipt of cancer treatment) is indistinguishable at the baseline (ie, cancer diagnosis where patients first become eligible).^16^^,^^17^ The main advantage of this cloning approach is that any early death or other study outcomes between the cancer diagnosis and initiation of chemotherapy will not be ignored (inducing potential selection bias) or assigned to the no treatment group only (inducing potential immortal time bias); rather, these outcome events will be counted in both treatment arms (because these are compatible with the definition of both treatment strategies) to avoid selection bias or immortal time bias.^18^ Briefly, we created a dataset with 2 copies of each eligible individual (ie, cloning) and assigned each of the replicates to one of the treatment strategies at the start of follow-up (T_0_) (ie, date of the diagnosis of cancer). We assessed whether patients adhered to their assigned treatment strategy at weekly intervals; patients were censored if and when their actual treatment deviated from their assigned treatment strategy, thereby ensuring that patients followed their assigned strategy. Inverse probability of censoring weight, calculated based on all the measured covariates, was used to account for the selection bias from artificial censoring. Details of the clone-censor-weight approach for emulating the target trial are presented in Supplemental Method 1.

Baseline characteristics are presented as the mean ± SD for continuous variables and as number and percentage for categorical variables. Because of the cloning step, all baseline characteristics were the same between fluoropyrimidine-treated and nontreated cohorts. The patient characteristics were remeasured after the grace period. We addressed missing data by creating an "unknown" category for variables with missing values, including ethnicity (3.7%), route of diagnosis (1.9%), and tumor grade (22.0%).

We estimated the effect of fluoropyrimidine on all-cause mortality and cardiovascular outcomes using adjusted, weighted pooled logistic regression at weekly intervals, including an indicator for treatment strategy, time (in its linear and quadratic terms), and their interactions to allow for nonproportional hazards. In addition to the weight, we also standardized the model-derived estimated values to the distribution of the baseline covariates. These standardized estimates were the predicted probabilities for survival from study outcomes on each treatment strategy at each time. This time-discrete survival probability was used to calculate the absolute risk at each time, and to plot the standardized, weighted cumulative incidence curves. We estimated the 1-year (52-week) absolute risks, risk differences (RDs), and risk ratios (RRs) with 95% CIs using a nonparametric bootstrap of 300 samples from the main analysis and 200 samples for secondary analyses. We also approximated HRs using ORs from the pooled logistic regression without the interaction terms between treatment indicator and time, and 95% CIs with the robust variance estimator, given that the outcome is rare during each weekly follow-up interval.^19^

Data cleaning was performed with R version 4.3.1 (R Foundation for Statistical Computing) and the clone-censor weight analysis was performed with SAS version 9.4 (SAS Institute).

### Competing risk event

In the analysis of cardiovascular outcomes, we consider noncardiovascular death as the competing risk event. In brief, we used a cause-specific model and censored patients at noncardiovascular death. We applied an inverse probability weight for death based on all baseline covariates to address the issue of artificial censoring. The details and rationale for choosing the current approach for handling the competing risk event are provided in Supplemental Method 2.

### Subgroup and sensitivity analysis

We conducted several prespecified subgroup and sensitivity analyses. The rationale for each analysis is explained in Supplemental Method 3. Analysis was repeated using cohorts stratified by pre-existing CVDs, age, sex, cancer site, and stage. In particular, stage II/III and stage IV cancers were analyzed separately due to key differences in their clinical characteristics, treatment approaches, and prognostic outcomes for both the survival and cardiovascular endpoints. We also analyzed mortality and cardiovascular events as a composite outcome to address the issue of competing risk from mortality. An additional analysis was performed excluding patients with missing data for performance status (PS) and with baseline PS of 4, and added PS as an additional regressor in weight calculations and outcome models. Further analyses extending the grace period from 8 weeks to 12 and 16 weeks were performed. We truncated the weight at the 99.5th percentile rather than the 99th. We further added a cubic term of the time into the model to allow more flexibility. We also performed the analysis with an additional outcome, nonspecific chest pain (ICD-10 codes R07.3 and R07.4). A negative control outcome of age-related cataract was used to quantitatively evaluate residual confounding. we explored the 1-year event rate for each study endpoint in patients with baseline coronary artery disease. Finally, we explore long-term outcomes up to 5 years after the cancer diagnosis.

## Results

### Patient characteristics

**Figure 1:**
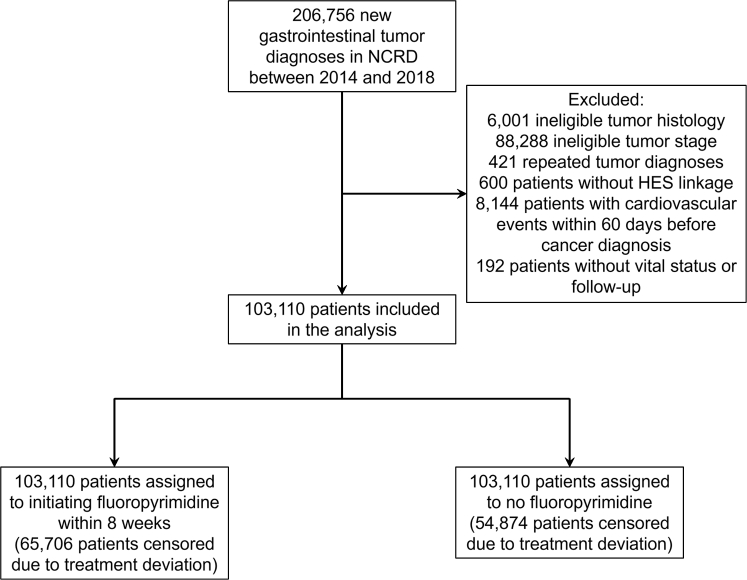
Selection of Patients From NCRD for the Target Trial Emulation There were 206,756 patients with new gastrointestinal tumor diagnosed between 2014 and 2018 in the National Cancer Registration Dataset (NCRD) database. After exclusion, 103,110 patients were eligible for the trial emulation. All patients were cloned and assigned with both treatment strategies. HES = Hospital Episode Statistics.

The cohort included 103,110 patients (Figure 1). Esophageal cancer, gastric cancer, and colorectal cancer was diagnosed in 21,392 (20.8%), 13,311 (12.9%), and 68,407 (66.3%) patients, respectively. The mean age of this study cohort was 69.7 ± 12.7 years. After censoring patients who died within the grace period of 8 weeks, there were 89,990 patients that remained in the analysis on week 9: 25,401 (28.2%) patients in the fluoropyrimidine and 64,589 (71.8%) patients in the no fluoropyrimidine cohorts. Patients who received fluoropyrimidines were younger and with fewer comorbidities, with esophageal and stage IV cancers more commonly represented than in those who did not receive fluoropyrimidines (Table 1). The inverse probability weights were calculated separately for each study outcome, and the distribution of the weights is presented in Supplemental Table 3 for the death outcome and Supplemental Table 4 for the cardiovascular events outcome.

**Table 1:** Baseline Characteristics of the Study Cohorts, Before and After the Grace Period

	All Patients (At Baseline Before the Grace Period) (N = 103,110)	Fluoropyrimidine (After the Grace Period) (n = 25,401)^a^	No Fluoropyrimidine (After the Grace Period) (n = 64,589)^a^
Age, y	69.7 ± 12.7	63.5 ± 11.6	71.0 ± 12.3
Male	61,517 (59.7)	16,234 (63.9)	37,632 (58.3)
Ethnicity			
White	93,893 (91.1)	23,147 (91.1)	59,006 (91.4)
Asian	2,223 (2.2)	569 (2.2)	1,454 (2.3)
Black	1,737 (1.7)	455 (1.8)	1,075 (1.7)
Mixed	380 (0.4)	107 (0.4)	231 (0.4)
Others	1,098 (1.1)	337 (1.3)	655 (1.0)
Unknown	3,779 (3.7)	786 (3.1)	2,168 (3.4)
CCI	0.6 ± 1.1	0.3 ± 0.8	0.6 ± 1.2
IMD quintiles			
1 (most deprived)	18,996 (18.4)	4,341 (17.1)	11,844 (18.3)
2	19,928 (19.3)	4,827 (19.0)	12,454 (19.3)
3	21,262 (20.6)	5,366 (21.1)	13,229 (20.5)
4	21,829 (21.2)	5,463 (21.5)	13,798 (21.4)
5 (least deprived)	21,095 (20.5)	5,404 (21.3)	13,264 (20.5)
Tumor site			
Esophageal	21,392 (20.8)	7,004 (27.6)	11,605 (18.0)
Gastric	13,311 (12.9)	3,634 (14.3)	6,963 (10.8)
Colorectal	68,407 (66.3)	14,763 (58.1)	46,021 (71.3)
Tumor stage			
II	6,281 (6.1)	1,706 (6.7)	4,287 (6.6)
III	49,619 (48.1)	10,859 (42.8)	37,087 (57.4)
IV	47,210 (45.8)	12,836 (50.5)	23,215 (35.9)
Tumor grade			
G1	2,068 (2.0)	588 (2.3)	1,356 (2.1)
G2	49,233 (47.7)	12,397 (48.8)	34,139 (52.9)
G3	28,856 (28.0)	8,130 (32.0)	16,865 (26.1)
G4	234 (0.2)	61 (0.2)	124 (0.2)
Unknown	22,719 (22.0)	4,225 (16.6)	12,105 (18.7)
Route of diagnosis			
Emergency presentation	23,817 (23.1)	3,666 (14.4)	12,492 (19.3)
GP referral	20,391 (19.8)	4,009 (15.8)	14,647 (22.7)
Inpatient elective	4,913 (4.8)	1,100 (4.3)	3,450 (5.3)
Other outpatient	5,570 (5.4)	1,229 (4.8)	3,834 (5.9)
Screening	4,935 (4.8)	924 (3.6)	3,973 (6.2)
2-wk wait	41,569 (40.3)	14,140 (55.7)	24,802 (38.4)
Unknown	1,915 (1.9)	333 (1.3)	1,391 (2.2)
Previous hospitalization			
Hypertension	16,119 (15.6)	1,903 (7.5)	10,961 (17.0)
Diabetes mellitus	5,652 (5.5)	631 (2.5)	3,866 (6.0)
COPD	3,775 (3.7)	329 (1.3)	2,546 (3.9)
CKD	2,281 (2.2)	118 (0.5)	1,527 (2.4)
CAD	12,027 (11.7)	1,595 (6.3)	8,311 (12.9)
Cardiac arrest	307 (0.3)	17 (0.1)	148 (0.2)
Cardiac arrhythmia	9,305 (9.0)	957 (3.8)	6,257 (9.7)
HF and cardiomyopathy	4,107 (4.0)	298 (1.2)	2,738 (4.2)
PVD	2,205 (2.1)	287 (1.1)	1,466 (2.3)
Pericarditis	409 (0.4)	62 (0.2)	251 (0.4)
Stroke	2,742 (2.7)	255 (1.0)	1,821 (2.8)
Valvular disease	3,649 (3.5)	376 (1.5)	2,525 (3.9)
VTE	3,476 (3.4)	439 (1.7)	1,932 (3.0)

Values are mean ± SD or n (%).

CAD = coronary artery disease; CCI = Charlson Comorbidity Index; CKD = chronic kidney disease; COPD = chronic obstructive pulmonary disease; GP = general practitioner; HF = heart failure; IMD = Index of Multiple Deprivation; PVD = peripheral vascular disease; VTE = venous thromboembolism.

a Patients in the fluoropyrimidine group (n = 25,401) and in the no fluoropyrimidine group (n = 64,589) are the patients who have already received fluoropyrimidine treatment or have not received fluoropyrimidine by the end of week 8, respectively. The patients who remained in the no fluoropyrimidine group may still have received fluoropyrimidine treatment during the remaining course of follow-up (and were censored at the fluoropyrimidine treatment). Patients who were censored or experienced outcomes by completion of the grace period (8 weeks from diagnosis) were excluded from this cohort. The total number of patients remaining in the analysis after 8 weeks therefore does not equate to the total number of patients at baseline.

### Fluoropyrimidine treatment and the risk of all-cause mortality

**Figure 2:**
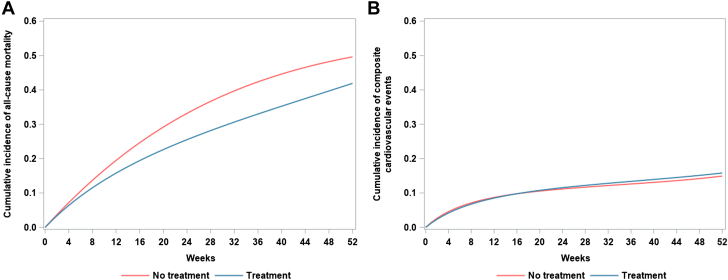
Weighted, Standardized Cumulative Incidence Curves Graphs showing weighted, standardized cumulative incidence curves for (A) all-cause mortality and (B) composite cardiovascular events, comparing patients on fluoropyrimidine vs no fluoropyrimidine.

**Figure 3:**
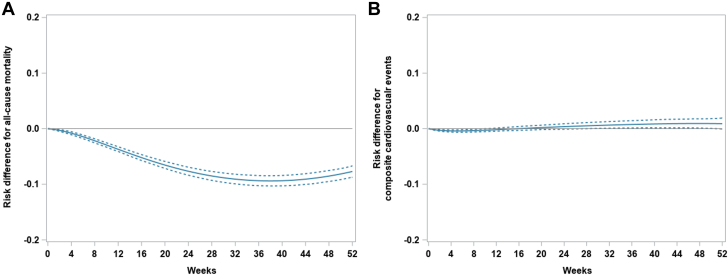
1-Year Absolute Risk Differences With 95% CIs Graphs showing 1-year absolute risk differences with 95% CIs from calculated weighted, standardized cumulative incidence for (A) all-cause mortality and (B) composite cardiovascular events, comparing patients on fluoropyrimidine vs no fluoropyrimidine.

There were 21,110 and 31,104 deaths in the fluoropyrimidine and no fluoropyrimidine cohorts, respectively. The absolute risk of death at 1 year was 41.9% (95% CI: 41.1% to 42.8%) for the fluoropyrimidine cohort and 49.6 (95% CI: 49.1% to 50.3%) for the no fluoropyrimidine cohort. The 1-year absolute RD in mortality was −7.7% (95% CI: −8.7% to −6.7%); the RR was 0.85 (95% CI: 0.83 to 0.86) (Figures 2A and 3A).

To show the average risk over the entire follow-up period, we also estimated HRs of fluoropyrimidine vs no fluoropyrimidine on mortality. The HRs were 0.76 (95% CI: 0.75 to 0.77) without any adjustments, 0.81 (95% CI: 0.78 to 0.84) after weighting, and 0.70 (95% CI: 0.68 to 0.73) after weighting and standardization (fully adjusted) (Table 2).

**Table 2:** Risks for All-Cause Mortality and Composite Cardiovascular Events Comparing Patients Receiving Fluoropyrimidines vs No Fluoropyrimidines

Treatment	Patients	Patient-Years	Outcomes	1-Year Absolute Risk (%) (95% CI)	1-Year Risk Difference (%) (95% CI)	1-Year Risk Ratio (95% CI)	HR (95% CI)
All-cause mortality							
Fluoropyrimidine	103,110	33,178	21,110	41.9 (41.1 to 42.8)	−7.7 (−8.7 to −6.7)	0.85 (0.83 to 0.86)	0.70 (0.67 to 0.73)
No fluoropyrimidine	103,110	37,657	31,104	49.6 (49.1 to 50.3)	Reference	Reference	Reference
Composite cardiovascular events							
Fluoropyrimidine	103,110	31,281	8,287	15.8 (15.0 to 16.6)	0.9 (0.0 to 1.9)	1.07 (1.00 to 1.13)	1.07 (1.00 to 1.16)
No fluoropyrimidine	103,110	34,228	9,873	14.9 (14.4 to 15.4)	Reference	Reference	Reference

### Fluoropyrimidine treatment and risk of cardiovascular events

There were 8,287 and 9,873 cases of composite cardiovascular events in the fluoropyrimidine and no fluoropyrimidine cohorts, respectively. The 1-year absolute risk of composite cardiovascular events was 15.8% (95% CI: 15.0% to 16.6%) for the fluoropyrimidine cohort and 14.9% (95% CI: 14.4% to 15.4%) for no fluoropyrimidine cohort. The 1-year RD for cardiovascular events was 0.9% (95% CI: 0.0% to 1.9%) and the 1-year RR was 1.07 (95% CI: 1.00 to 1.13) for the fluoropyrimidine vs no fluoropyrimidine cohorts (Figures 2B and 3B). The HRs of fluoropyrimidine vs no fluoropyrimidine on cardiovascular events were 0.87 (95% CI: 0.86 to 0.89) without any adjustments, 0.97 (95% CI: 0.90 to 1.03) after weighting, and 1.07 (95% CI: 1.00 to 1.16) after weighting and standardization (Table 2).

For individual cardiovascular events, there was a higher risk of cardiac arrhythmia and cardiac arrest for fluoropyrimidine treatment. The 1-year RD for fluoropyrimidine vs no fluoropyrimidine was higher for cardiac arrhythmia (RD: 0.82%; 95% CI: 0.11% to 1.58%) and cardiac arrest (RD: 0.34%; 95% CI: 0.13% to 0.47%). There was no difference for ACS (RD: −0.07%; 95% CI: −0.63 to 0.51%), HF and cardiomyopathy (risk difference: −0.03%; 95% CI: −0.49% to 0.42%), cardiac intervention (RD: 0%; 95% CI: −0.06% to 0.06%), or cardiac death (RD: 0.16%; 95% CI: −0.22% to 0.47%) (Supplemental Figure 1, Supplemental Table 5).

### Subgroup and sensitivity analyses

The detailed results for the subgroup and sensitivity analyses are presented in the Supplemental Tables 6 to 15. A lower risk of all-cause mortality was observed consistently in the fluoropyrimidine group across all patient subgroups and sensitivity analyses. Among patients with stage II/III cancer, the 1-year RR for mortality was 0.71 (95% CI: 0.67 to 0.75); among patients with stage IV cancer, the 1-year RR for mortality was 0.89 (95% CI: 0.87 to 0.91) (Supplemental Table 6). However, fluoropyrimidine treatment was associated with an increased risk of cardiovascular events in patients without baseline cardiovascular events, stage II/III cancer, and gastric cancer. The RD ranged from 0.7% to 4.5%. Among patients with stage II/III cancer, the 1-year RR for cardiovascular events was 1.09 (95% CI: 1.03 to 1.18); among patients with stage IV cancer, the 1-year RR for cardiovascular events was 1.00 (95% CI: 0.91 to 1.11) (Supplemental Table 7).

There was a lower risk of the composite outcome of all-cause mortality and cardiovascular events with fluoropyrimidine treatment (1-year RR: 0.89) (Supplemental Table 8, Supplemental Figure 2). Adjusting for performance status (Supplemental Table 9), varying grace period (12 and 16 weeks), truncating the inverse probability of censoring weights at different percentiles, or adding a cubic term of time did not materially alter the results (Supplemental Tables 10 to 14). There was a tendency toward a higher risk of nonspecific chest pain with fluoropyrimidine treatment (RD: 0.57%; 95% CI: −0.01% to 1.14%; RR: 1.22; 95% CI: 1.00 to 1.48) (Supplemental Figure 3; Supplemental Table 15). For the negative control outcome, there was no difference in the risk of cataract between treatment groups, with a point estimate very close to 1 after weighting and adjustment (HR: 0.97; 95% CI: 0.61 to 1.54), while the unadjusted HR was 0.48 (95% CI: 0.41 to 0.57). The 1-year absolute risk, RD, and RR for individual study endpoints in patients with baseline ACS between groups are presented in Supplemental Table 16. The RD for all-cause mortality was similar to the main analysis (−8.6%), while the RD for composite cardiovascular events was 0.0%; for each cardiovascular event endpoint, the RD was lower than 1.0%. Last, when we extended follow-up period to 5-year after the cancer diagnosis, we observed consistent survival benefit (5-year RR: 0.87) but no increased risk of cardiovascular events (5-year RR: 0.93) (Supplemental Table 17).

## Discussion

In this large nationwide observational study explicitly emulating a target pragmatic trial, we found a small increase in absolute risk of cardiovascular events (0.9%) in patients with gastrointestinal cancer treated with fluoropyrimidines compared with those receiving alternative therapies. However, this was significantly outweighed by an almost 8% absolute risk reduction in all-cause mortality at 1 year. This represents an almost 8-fold difference between the benefit from reduction in the risk of death from cancer with fluoropyrimidine therapy, and the small increased risk of a cardiovascular event (Central Illustration). Cardiovascular events overall were common (15.8% in fluoropyrimidine group and 14.9% in no fluoropyrimidine group) across both treatment groups, and ACSs, coronary interventions, and HF were similar irrespective of treatment strategy. However, rates of arrhythmia and cardiac arrest were modestly higher in those receiving fluoropyrimidines. Even in patients with pre-existing CVD, there were no differences in overall cardiovascular event rates between treatment groups.

**Central Illustration:**
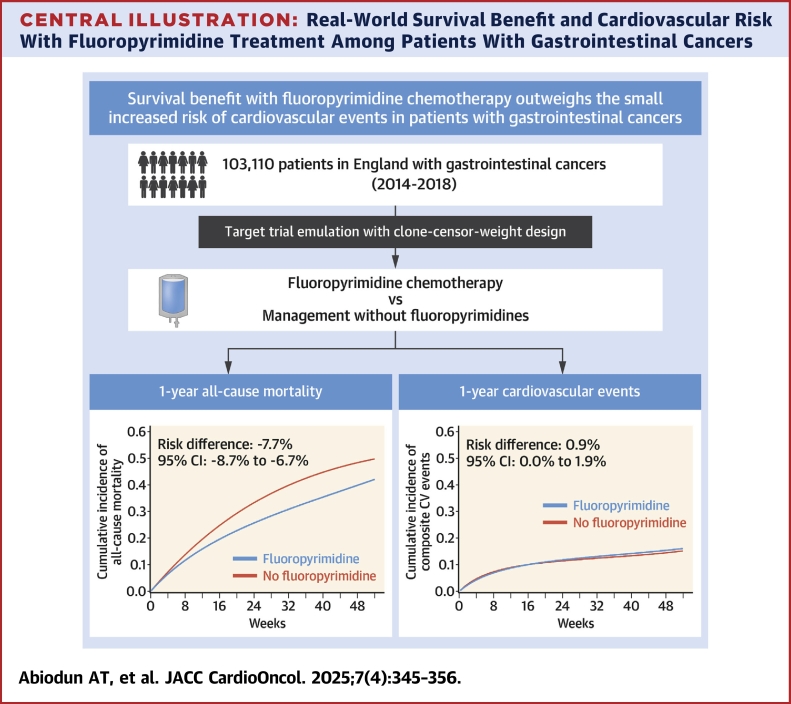
Real-World Survival Benefit and Cardiovascular Risk With Fluoropyrimidine Treatment Among Patients With Gastrointestinal Cancers Real-world observational data of 103,110 patients with gastrointestinal cancers from England were used to emulate a target trial to evaluate the survival benefit and cardiovascular risk with fluoropyrimidine treatment. There was a 7.7% lower absolute risk difference for all-cause mortality and a 0.9% higher absolute risk difference for cardiovascular events at 1 year after the cancer diagnosis comparing fluoropyrimidine vs no fluoropyrimidine.

This is the first large-scale epidemiological study directly comparing cardiovascular events in patients with gastrointestinal cancers treated with fluoropyrimidines with those not receiving fluoropyrimidines. Fluoropyrimidine chemotherapy remains the first-line treatment for many gastrointestinal cancers;^1^^,^^14^^,^^20^ however, previous analysis of this same linked national registry VICORI dataset showed that patients with established CVD are 27% less likely to receive these treatments despite tumor eligibility, perhaps due to concerns regarding cardiotoxicity.^21^ Reported incidence of cardiovascular events with fluoropyrimidines varies widely, from 0% to 35% in the literature,^22^ with coronary spasm leading to ACSs widely recognized to be a common complication. However, these reported data have substantial heterogeneity due to varied study population, follow-up time, and definition of the cardiotoxicity.^22^ Initial RCTs of fluoropyrimidines were not powered to assess for cardiovascular events, and in 1 review of 16 phase II and III clinical trials involving fluoropyrimidines between 2002 and 2017, only 44% of studies specifically collected data on cardiac ischemia/infarction and patients with pre-existing CVD were commonly excluded.^23^

We included comprehensive national data, thereby presenting real-world clinical information, with the results demonstrating the high rates of pre-existing CVD and cardiovascular risk factors. This aspect reflects a population that would often be excluded from clinical trials due to their cardiovascular comorbidities or risk factor.^24^ By incorporating these patients, our study offers insights into outcomes for a broader and more representative patient population, enhancing the generalizability and clinical relevance of the findings. We observed rates of all cardiovascular events in the first year after cancer diagnosis were high, irrespective of treatment strategy. This highlights the importance both of ensuring that appropriate cancer controls arms are used to enhance exchangeability between comparison groups in terms of the cardiovascular risk, and the challenges of assessing the incremental impact of fluoropyrimidine treatment above the high background event rates.

The risk of fluoropyrimidine-associated cardiotoxicity has been investigated in prior cohort studies.^25^^,^^26^ Shanmuganathan et al^25^ used Danish national data to show a nonsignificant difference in myocardial infarction at 12 months; however, overall events were low (0.9% vs 0.6%; *P* = .051). The second study used population data from Hong Kong and found an increased risk of composite cardiovascular events but was predominantly driven by stroke events.^26^ Patients included in these studies differed significantly from the multiethnic cohort included in this current study. Both were also limited by the lack of granular cancer diagnosis information and used noncancer control subjects. This strongly limits causal interpretations, regardless of adjustments made for confounding variables. In contrast, we compared patients with similar types of cancer treated with fluoropyrimidine chemotherapy or not, thereby designed to mitigate selection bias arising from the inherent cardiovascular risks associated with the cancer itself.^27^^,^^28^

In our study, we found that of all potential cardiovascular events, fluoropyrimidine-treated patients are at a small increased absolute risk of cardiac arrhythmias and cardiac arrest compared with their untreated counterparts. In a recent systematic review and meta-analysis that included 211 studies comprising of a total 63,186 patients, cardiac arrhythmias were found to be the second most common manifestation of fluoropyrimidine-associated cardiotoxicity at 1.85%, the first being coronary disorders.^29^ The definitions of arrhythmia vary greatly across studies, with higher incidence rates (up to 20%) previously noted in some Asian studies,^30^^,^^31^ although manifestations such as sinus bradycardia (which often do not require treatment) have been included as cardiotoxic events in these studies. We did not include this endpoint in our study. Our sensitivity analysis showed that more fluoropyrimidine-treated patients experienced nonspecific chest pain during treatment compared with their untreated counterparts, but the difference was not significant.

Our findings confirm that all-cause mortality is significantly lower in patients treated with fluoropyrimidines, with similar effect sizes with the previous RCTs.^32^ In patients with resectable esophagogastric tumors, perioperative fluoropyrimidine-based chemotherapy in addition to surgery reduced death by 25%,^24^ as compared with 23% for esophageal cancer and 20% for gastric cancer in our study.

### Study strengths

This is the first study of its kind using a target trial emulation approach to compare fluoropyrimidine-treated patients with untreated patients in terms of all-cause mortality and cardiovascular toxicity. As a large, prospective RCT is unlikely to be available in the near future to answer this question, our study utilizing observational data provides an alternative research approach to answer the current question. The large sample size ensured ample statistical power to determine whether fluoropyrimidine treated patients are at increased risk of cardiovascular toxicity to a high degree of confidence. Utilizing advanced analytic methods, we have been able to produce a comprehensive analysis with both absolute and relative risks given, which may help better inform oncology clinicians when counseling and consenting patients on fluoropyrimidine and cardiovascular risks. Finally, our findings on the survival benefits of fluoropyrimidine being consistent with the previous evidence and the null association with the negative control outcome support the validity of our analysis.

### Study limitations

First, in line with all causal inference models, our study has assumptions, namely the exchangeability, consistency, and positivity.^33^ However, there might be possible violations to the assumptions, for example, poor exchangeability due to residual confounding. To put this into context, the VICORI data do not have linkage to primary care records; therefore, data recorded only in the primary care setting cannot be determined, including pre-existing use of cardiovascular medications. These variables could be important confounders. For the measured comorbidities, we also did not have information on severity. Because younger and healthier patients were more likely to be treated with fluoropyrimidines in our cohort, the current estimates from this study are likely underestimating the cardiotoxicity of fluoropyrimidines. However, our negative control analysis shows the magnitude of the residual confounder is small and unlikely to impact our results significantly.^34^ Second, ascertainment of treatments may not be complete, although previous studies reported 85% to 95% completeness of the SACT data in chemotherapy records.^35^^,^^36^ Data on chemotherapy dosage and number of treatment cycles are incomplete, thereby preventing us from including these in the analysis. However, the estimates of mortality from our target trial are close to the estimates from previous RCTs and observational studies.^24^^,^^32^^,^^37^ Coronary artery vasospasm is one of the most frequent presentations of fluoropyrimidine cardiotoxicity; however, it does not have an independent ICD-10 classification. Therefore, we investigated ischemic heart diseases as a composite group, which includes angina with documented spasm (I20.1) and ACS.^38^ Pretreatment screening for dihydropyrimidine dehydrogenase mutations was only mandated in the United Kingdom in 2020 for fluoropyrimidine-treated patients; therefore, results were not available for our cohort.^39^

## Conclusions

The incremental risk of cardiovascular events during fluoropyrimidine treatment is small when compared with an almost 8-fold reduction in all-cause mortality in patients with gastrointestinal cancers. Irrespective of treatment strategy, cardiovascular events were high, with 1 in 7 patients experiencing an event within the 12-month follow-up period. However, there was no increased risk of cardiovascular death, ACS, HF hospitalization, or requirement for cardiac intervention, even for patients with pre-existing CVD. There was a small increased risk of cardiac arrhythmias and cardiac arrest. The substantial survival benefit from fluoropyrimidines should be weighed against this modest cardiovascular concern by oncologists considering alternative therapies for patients with gastrointestinal cancers.

Perspectives **COMPETENCY IN MEDICAL KNOWLEDGE:** In this national cohort study of 103,110 patients with stage II to IV esophageal or gastric cancer and stage III or IV colorectal cancer, patients receiving fluoropyrimidine treatment had only a 0.9% higher absolute risk of acute cardiovascular events over 12 months, when compared with patients not treated with fluoropyrimidines, despite a 7.7% lower absolute risk difference in death. Overall rates of cardiovascular events were high (1 in 7) across all patients, irrespective of fluoropyrimidine treatment.

**TRANSLATIONAL OUTLOOK:** The findings suggest that the survival benefit of fluoropyrimidine-based chemotherapy greatly outweighs the risk of cardiovascular events in patients with gastrointestinal cancer, even in those with a prior history of coronary artery disease.

## Funding Support and Author Disclosures

This study was jointly funded by Cancer Research UK (C53325/A21134) and the British Heart Foundation (SP/16/5/32415). Dr Abiodun is funded by British Heart foundation Clinical Research Training Fellowship (FS/CRTF/21/24134). Professor Manisty is supported directly and indirectly from the NIHR Biomedical Research Centers at the University College London Hospitals and Barts Health NHS Trusts. Professor Manisty is a co-founder and board member of MyCardium AI. Professor Adlam has received research funding from Abbott Vascular to support a clinical research fellow; received funding from AstraZeneca for unrelated research; served as a consultant for General Electric to support research funds; received royalties from Elsevier for the *ECG Made Easy*, *ECG Made Practical*, and *ECG Problems* books; and holds the following unrelated patents (EP3277337A1, PCT/GB2017/050877, UK patent application number 2211616.4). Dr Slater has received consulting fees from the Pfizer Cachexia board and EISAI. All other authors have reported that they have no relationships relevant to the contents of this paper to disclose.
