# Supplementary material for: Fluoropyrimidine Chemotherapy and the Risk of Death and Cardiovascular Events in Patients With Gastrointestinal Cancer
Source: JACC CardioOncol. 2025 Apr 8;7(4):345–56. doi: 10.1016/j.jaccao.2025.01.019 (PMC12228134; doi:10.1016/j.jaccao.2025.01.019)
Supplement: Supplemental Material [file mmc1.docx]

Fluoropyrimidine chemotherapy and the risk of death and cardiovascular events in patients with gastrointestinal cancer: a cohort study

**SUPPLEMENTARY MATERIAL**

Contents

Supplemental [Table 1. Brief specifications for the target trial and the target trial emulation using observational data. 3](#_Toc187317155)

Supplemental [Table 2. Variable definitions and sources. 6](#_Toc187317156)

Supplemental [Methods 1. Details of the clone-censor-weight analysis. 8](#_Toc187317157)

Supplemental [Methods 2. Competing risk event and target estimand. 13](#_Toc187317158)l

Supplemental [Methods 3. Rationales for the subgroup and sensitivity analysis. 15](#_Toc187317159)

Supplemental [Table 3. Inverse probability weight distribution for death outcome. 17](#_Toc187317160)

Supplemental [Table 4. Inverse probability weight distribution for composite cardiovascular events outcome. 18](#_Toc187317161)

Supplemental [Figure 1. Weighted, standardized cumulative incidence curves for (A) acute coronary syndrome; (B) heart failure and cardiomyopathy; (C) cardiac intervention; (D) cardiac arrhythmia; (E) cardiac arrest; (F) cardiac death comparing patients receiving fluoropyrimidine versus no fluoropyrimidine treatment. 19](#_Toc187317162)

Supplemental [Table 5. One-year risk differences, risk ratios, and hazard ratios for individual cardiovascular events comparing patients on fluoropyrimidine versus no fluoropyrimidine. 20](#_Toc187317163)

Supplemental [Table 6. One-year absolute risks, risk differences, risk ratios, and hazard ratios for all-cause mortality comparing patients on fluoropyrimidine versus no fluoropyrimidine, stratified by subgroups. 22](#_Toc187317164)

Supplemental [Table 7. One-year absolute risks, risk differences, risk ratios, and hazard ratios for composite cardiovascular events comparing patients on fluoropyrimidine versus no fluoropyrimidine, stratified by subgroups. 24](#_Toc187317165)

Supplemental [Table 8. Sensitivity analysis: composite outcome of all-cause mortality and cardiovascular events and the point estimates for one-year absolute risk, risk difference, risk ratio, and hazard ratio. 26](#_Toc187317166)

Supplemental [Table 9. Distribution of performance status of the study cohorts, before and after the grace period. 27](#_Toc187317167)

Supplemental [Table 10. Sensitivity analysis: performance status being controlled and the point estimates for one-year absolute risk, risk difference, risk ratio, and hazard ratio for all-cause mortality and composite cardiovascular events. 28](#_Toc187317168)

Supplemental [Table 11. Sensitivity analysis: 12-week grace period and the point estimates for one-year absolute risk, risk difference, risk ratio, and hazard ratio for all-cause mortality and composite cardiovascular events. 29](#_Toc187317169)

Supplemental [Table 12. Sensitivity analysis: 16-week grace period and the point estimates for one-year absolute risk, risk difference, risk ratio, and hazard ratio for all-cause mortality and composite cardiovascular events. 30](#_Toc187317170)

Supplemental [Table 13. Sensitivity analysis: weight truncation at 99.5^th^ percentile and the point estimates for one-year absolute risk, risk difference, risk ratio, and hazard ratio for all-cause mortality and composite cardiovascular events. 31](#_Toc187317171)

Supplemental [Table 14. Sensitivity analysis: time added in its linear, quadratic, and cubic terms and the point estimates for one-year absolute risk, risk difference, risk ratio, and hazard ratio for all-cause mortality and composite cardiovascular events. 32](#_Toc187317172)

Supplemental [Table 15. Sensitivity analysis: one-year risk differences, risk ratios, and hazard ratios for non-specific chest pain comparing patients on fluoropyrimidine versus no fluoropyrimidine. 33](#_Toc187317173)

Supplemental [Table 16. Exploratory analysis: one-year absolute risk, risk difference, and risk ratio of each study outcome comparing patients on fluoropyrimidine versus no fluoropyrimidine, among patients with baseline coronary artery disease. 34](#_Toc187317174)

Supplemental [Table 17. Exploratory analysis: five-year absolute risks, risk differences, risk ratios, and hazard ratios for all-cause mortality and composite cardiovascular events comparing patients on fluoropyrimidine and versus no fluoropyrimidine. 35](#_Toc187317175)

Supplemental [Figure 2. Weighted, standardized cumulative incidence curves for composite all-cause mortality and cardiovascular events comparing patients on fluoropyrimidine versus no fluoropyrimidine. 36](#_Toc187317176)

Supplemental [Figure 3. Weighted, standardized cumulative incidence curves for non-specific chest pain comparing patients on fluoropyrimidine versus no fluoropyrimidine. 37](#_Toc187317177)

[References 38](#_Toc187317178)

# Supplemental Table 1. Brief specifications for the target trial and the target trial emulation using observational data.

| **Component** | **Target trial specification** | **Target trial emulation** |
| --- | --- | --- |
| Eligibility criteria | Patients aged between 18 years and 100 years with a first diagnosis of primary gastrointestinal carcinoma (stage II to IV for esophageal and gastric cancer and stage III to IV for colorectal cancer) in England between 1^st^ January 2014 to 31st March 2018; no treatment of fluoropyrimidine before enrolment; no cardiovascular events within 60 days before enrolment. | As per the target trial.  We additionally exclude patients with missing tumor stages and patients who met the standard NCRAS exclusion criteria, patients without linkage to HES data, or patients with missing vital status or no follow-up. |
| ªTreatment strategies | 1. Receiving fluoropyrimidine chemotherapy-based regimen within 8 weeks after the cancer diagnosis, and patients can also receive other treatments as appropriate.  2. No fluoropyrimidine chemotherapy during follow-up, but patients can receive other treatments as appropriate. | As per the target trial. |
| Treatment assignment | Patients are randomly assigned to a strategy at baseline and will be aware of the strategy they have been assigned. | Randomization is emulated via the clone-censor-weight approach. |
| Outcomes | All-cause mortality.  Cardiovascular events include acute coronary events, cardiac intervention, arrhythmia, heart failure, cardiac arrest, and cardiovascular death. | As per the target trial. |
| Follow-up | For each patient, the follow-up starts at the enrolment (cancer diagnosis) and ends at the outcome, death, or 52 weeks after the baseline, whichever occurs first. | As per the target trial. |
| Causal contrasts | Intention-to-treat effect.  Per protocol effect. | Observational analogue of per-protocol effect. |
| Statistical analysis | Intention-to-treat analysis.  Per protocol analysis: patients are censored when they deviate from the assigned treatment strategy, i.e., if the patients on the fluoropyrimidine arm did not receive the treatment by the end of the 8-week, or if the patients on the no-fluoropyrimidine arm received the treatment during the follow-up. Selection bias introduced by artificial censoring is accounted for by using inverse probability of censoring weights that are calculated based on baseline covariates.  Competing risk from death is accounted by applying an inverse probability of censoring weight for death.  Pre-specified subgroup and sensitivity analyses:   1. Stratified by pre-existing CVD, age of 60 years, gender, cancer site, cancer stage (IV or II to III). 2. Non-specific chest pain as an additional endpoint. 3. Composite outcome of death and cardiovascular events | Only per-protocol effect analysis is conducted.  Same pre-specified subgroup and sensitivity analyses.  Additional sensitivity analyses for validating the emulation process:   1. In patients with performance status recorded and it is not 4. 2. Truncation of weight at 99.5%. 3. Age-related cataract as a negative control outcome. 4. Modify the treatment strategy from “receiving fluoropyrimidine chemotherapy-based regimen within 8 weeks after the cancer diagnosis” to “receiving fluoropyrimidine chemotherapy-based regimen within 12 and 16 weeks after the cancer diagnosis”. |

# Supplemental Table 2. Variable definitions and sources.

| **Variable** | **Definition and source*** |
| --- | --- |
| Age at cancer diagnosis | NCRD definition |
| Gender | NCRD definition, self-reported gender by patients |
| Ethnicity group | NCRD definition |
| Charlson Comorbidity index | NCRD definition |
| IMD quintiles | NCRD definition |
| Tumor site | NCRD definition, ICD-10 codes C15, C16, C18-C21 |
| Tumor stage | NCRD definition |
| Tumor grade | NCRD definition |
| Route of cancer diagnosis | NCRD definition |
| Hypertension | HES, ICD-10 codes I10 |
| Diabetes mellitus | HES, ICD-10 codes E11 |
| COPD | HES, ICD-10 codes J43, J44 |
| CKD | HES, ICD-10 codes N18.3-N18.5, D63.1, E10.2, E11.2, E12.2, E13.2, E14.2, I12, I13, N03-N05, Z94.0 |
| CAD | HES, ICD-10 codes I20-I25 |
|  | HES, OPCS-4 codes K40-K46 |
|  | MINAP, excluding diagnosis code 6, 8, 9 (unconfirmed diagnosis) |
|  | HES, OPCS-4 codes K40-K46, K49, K50, K75 |
|  | NAPCI, any record |
|  | NACSA, CABG=1 |
| VTE | HES, ICD-10 codes I80.1-I80.3, I26 |
| HF and cardiomyopathy | HES, ICD-10 codes I25.5, I42, I43, I50 |
|  | NHFA, any record |
| Valvular disease | HES, ICD-10 codes I01-I08, I34-I37 |
|  | NACSA, VALVE=1 |
| Cardiac arrhythmia | HES, ICD-10 codes I48, I49 |
| Stroke | HES, ICD-10 codes I60-I64 |
| PVD | HES, ICD-10 codes I73 |
| Cardiac arrest | HES, ICD-10 codes I46 |
| Pericarditis | HES, ICD-10 codes I30-I32 |
| ACS | HES, ICD-10 codes I20-I25 |
|  | HES, OPCS-4 codes K40-K46 |
|  | MINAP, excluding diagnosis code 6, 8, 9 (unconfirmed diagnosis) |
| Coronary intervention | HES, OPCS-4 codes K40-K46, K49, K50, K75 |
|  | NAPCI, any record |
|  | NACSA, CABG=1 |
| Cardiovascular disease | Any of CAD, VTE, HF and cardiomyopathy, valvular disease, cardiac arrhythmia, stroke, PVD, cardiac arrest, or pericarditis. |
| Death and cause of death | ONS, ICD-10 codes |
| Fluoropyrimidine treatment | SACT, 5-FU or capecitabine analysis group |
|  | AV treatment, 5-FU or capecitabine |
| Non-specific chest pain | HES, ICD-10 codes R07.3 and R07.4 |

*Further information on variable availability and definition in NCRD can be found in previously published data profile ^1^ .

# Supplemental Methods 1. Details of the clone-censor-weight analysis.

The clone-censor-weight design is a three-step approach that is commonly used to emulate target pragmatic trials using observational data. As we explained in the main text and in Table S1, the trial emulation has two steps. First, we designed a pragmatic target trial that would answer the causal question of interest; then, we emulated this using data from VICORI. As there are intrinsic differences between observational data in real world and data from a randomized controlled trial settings (mainly due to the lack of randomized treatment assignment at the cohort entry), we would need certain study designs to allow a close emulation of the target trial, e.g., clone-censor-weight. The main advantage of this approach is in reduction of bias when the treatment status (i.e., the receipt of cancer treatment) is indistinguishable at the baseline (i.e., cancer diagnosis where patients first become eligible).


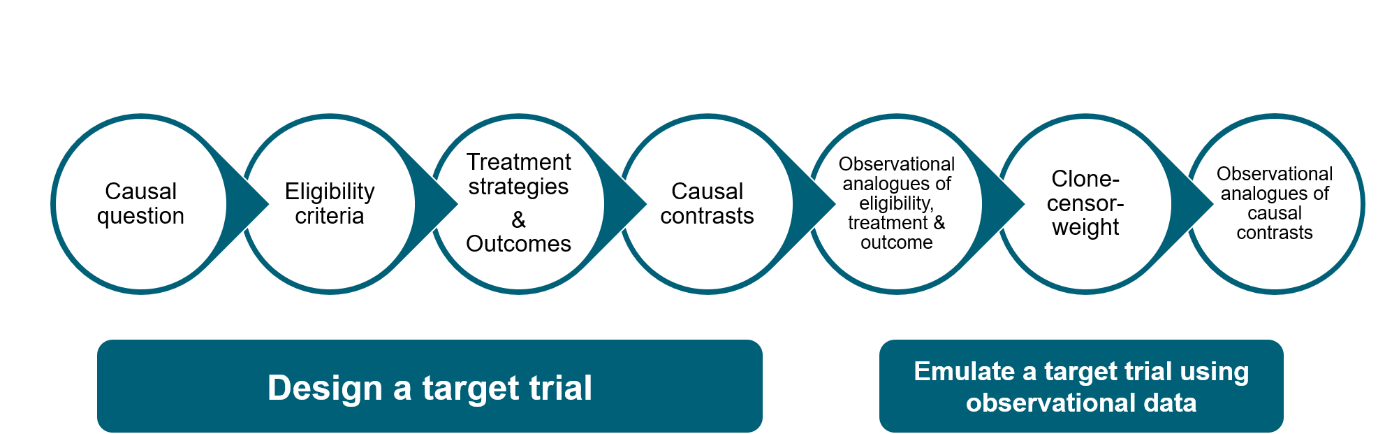


**Schema of trial emulation with clone-censor-weight approach.**

The technical details of the designs have been described elsewhere. Here we briefly introduce the application of the design in this study. The clone-censor-weight is a three-step approach, as illustrated below:


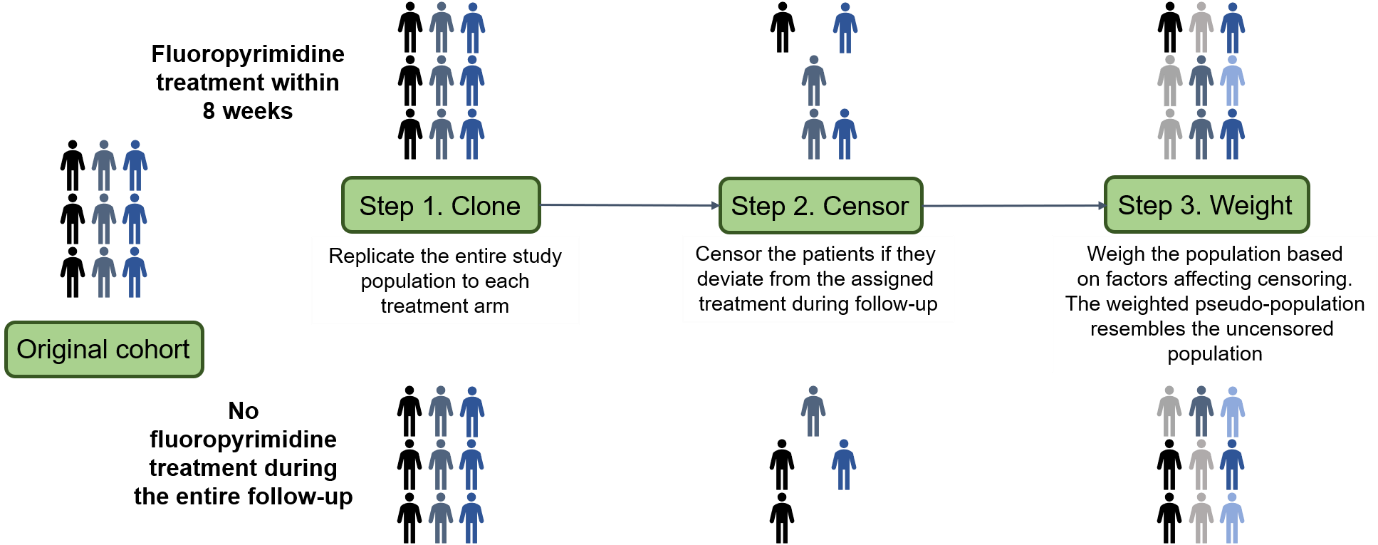


**Overview of the clone-censor-weight approach.**

We further explained each step in details:

**Step 1. Clone**

From the dataset that contains all study variables of all eligible patients, we simply replicate this dataset to *N* copies (*N* equals to the number of treatment strategies one would like to compare in the target trial, in this case, *N*=2). One copy is assigned to the “receiving fluoropyrimidine treatment” strategy and the other copy is assigned to the “no fluoropyrimidine treatment” strategy.

**Step 2. Censor**

Because the treatment assignment is hypothetical, patient replicates are likely to deviate from the assigned treatment strategy in real world. Therefore, we censor the patient replicates upon treatment deviation. That is, if a patient replicate is assigned to receiving fluoropyrimidine treatment strategy and has not done so within the grace period, they are censored after the grace period; if a patient replicate is assigned to no fluoropyrimidine treatment strategy but received the treatment at any time during the follow-up, they are censored at the treatment initiation.

The cloning and censoring steps are further illustrated with the diagram below, in which we used four hypothetical patient examples to illustrate the procedures.

**
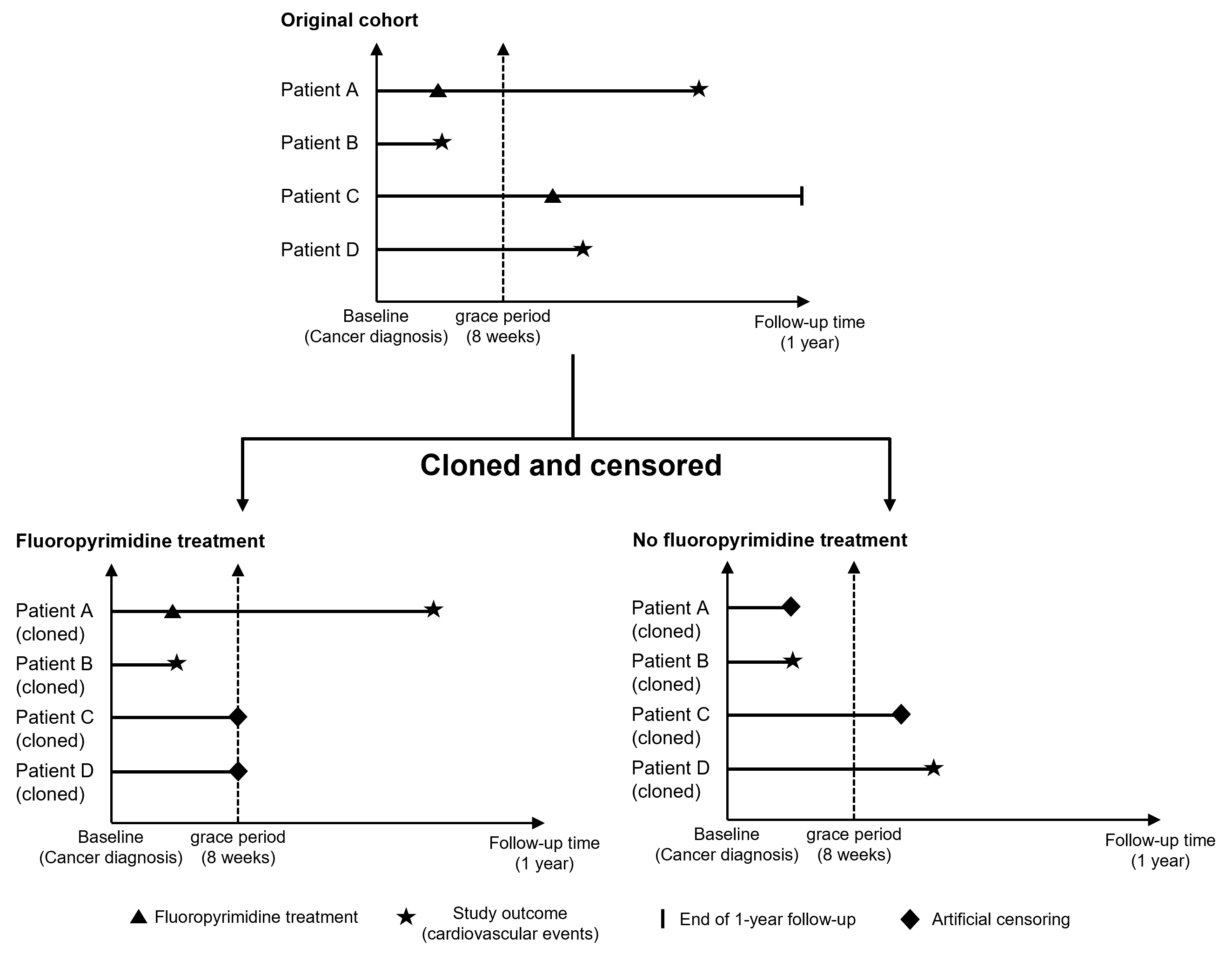
**

**Illustration of cloning and censoring design used in this study.**

Patient A received fluoropyrimidine within the 8-week grace period and experienced a study outcome (e.g., death) during follow-up.

Patient B had a study outcome within the grace period before receiving fluoropyrimidine.

Patient C received fluoropyrimidine after the grace period and did not experience an adverse outcome during the 1-year follow-up period therefore reached the end of follow-up.

Patient D had a study outcome after the grace period and had not received any fluoropyrimidine prior to this event.

All patients are cloned and assigned to both “fluoropyrimidine treatment within 8-week” and “no fluoropyrimidine treatment”.

Patient A assigned to fluoropyrimidine arm is compatible with the assigned strategy and is therefore not artificially censored and followed until the study outcome; Patient A assigned to no fluoropyrimidine arm was artificially censored at the fluoropyrimidine treatment.

Patient B is not censored in either treatment arm, and contributes the outcome to both treatment strategies.

Patient C assigned to fluoropyrimidine arm is artificially censored after the grace period; Patient C assigned to no fluoropyrimidine arm is artificially censored at the fluoropyrimidine treatment after the grace period.

Patient D assigned to fluoropyrimidine arm is artificially censored after the grace period; Patient D assigned to no fluoropyrimidine arm is not artificially censored and followed until the study outcome.

**Step 3. Weight**

Because the artificial censoring in step 2 can induce attrition bias, we correct the bias by building an inverse probability of censoring weight, based on the probability of a patient (replicate) of being uncensored at each time. To do so, we first calculate the probability of a patient receiving fluoropyrimidine treatment at each time interval based on their baseline characteristics.

This probability is calculated using logistic regression models. The models included the main effects between the treatment status and the predictors, which are all the measured covariates listed in Table 1. For the continuous variables, we assumed linear effects on the predicted treatment status. We fit two models, one for each treatment strategy arm, to allow treatment-covariate interactions. The first model, used for receiving fluoropyrimidine treatment arm, was restricted to time interval (weekly) from t0 to t7 (corresponding to the 8-week grace period in the main analysis and was changed to t11 and t15 in the sensitivity analyses with 12-week and 16-week grace period, respectively); the second model, used for the no fluoropyrimidine treatment arm used all observations from time interval t0 to t51. Then the inverse probability weight is built as

$$W_{t}^{A=1}=\frac{1}{1-\prod_{k=0}^{t} Pr(a_{k}=0|Y_{k-1}=0,D_{k-1}=0,\overline{a}_{k-1}=0,V)}$$

$$W_{t}^{A=0}=\frac{1}{\prod_{k=0}^{t} Pr(a_{k}=0|Y_{k-1}=0,D_{k-1}=0,\overline{a}_{k-1}=0,V)}$$

where Y=outcome, D=death, A=treatment assignment, a=treatment initiation, V=baseline characteristics, t=time interval since baseline

The denominator of the weights is the probability that a replicate remained on the assigned treatment strategy conditional on baseline characteristics. The weights created two pseudo-populations in which treatment initiation was independent of measured prognostic factors. We estimated the time-varying weights by fitting a pooled logistic model for the weekly probability of remaining uncensored, including variables for time (in its linear and quadratic terms) and the baseline covariates as mentioned.

The $W_{t}^{A=1}$is artificially kept as 1 between t0 and t7, and unchanged from t9 and the remaining follow-up time (in the main analysis and were changed in the sensitivity analyses with longer grace period). This is because during these time intervals, by definition, the artificial censoring cannot occur.

To avoid undue influence of outliers, all weights are truncated at the 99th percentile.

**Step 4. Analysis**

The replicated datasets with calculated weights are stacked and analyzed with a weighted pooled logistic regression model. The model further included all baseline covariates, a treatment indicator, time (in its linear and quadratic terms), and the interaction terms between time (in its linear and quadratic terms) and treatment indicator. This model predicts the risk of study outcome for each participant on each treatment strategy at each time interval. We can use these predicted risks to compute the population-average cumulative risk (and risk difference) of the study outcome on treatment strategies. This can be interpreted as the causal estimand that we aim to answer under the assumption of exchangeability, positivity, consistency, and no model misspecification.

# Supplemental Methods 2. Competing risk event and target estimand.

In the analysis of cardiovascular outcomes, we consider non-cardiovascular death as the competing risk event. The role of the competing risk by death is illustrated using a causal diagram below:


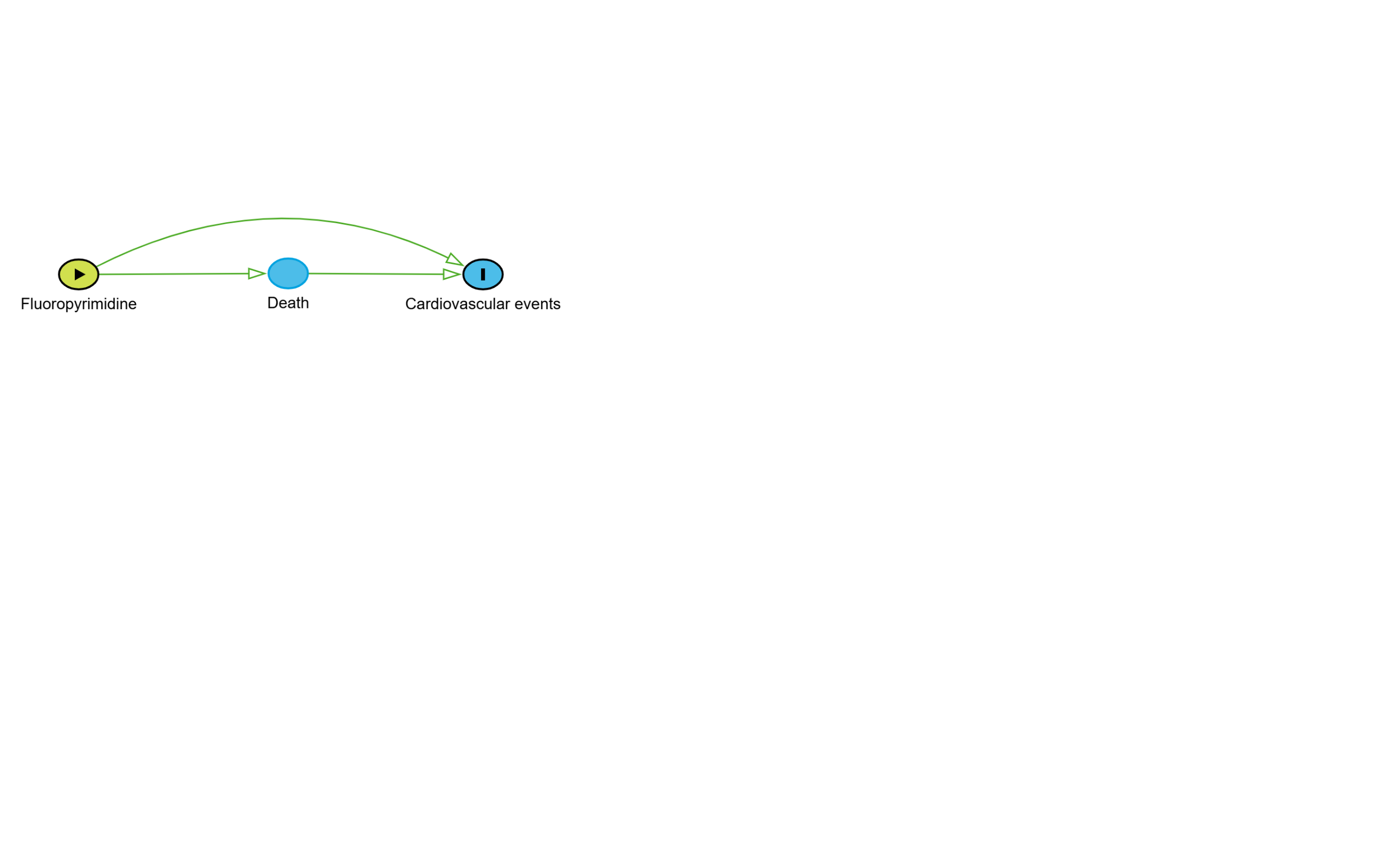


**A causal diagram demonstrating the effect of fluoropyrimidine treatment on cardiovascular events and role of competing risk by death**

In the current study, we handled the competing risk by censoring non-cardiovascular death in a cause-specific model. Because censoring due to death may introduce selection bias, we additionally calculate an inverse probability of death weight to address the common causes between death and cardiovascular events, based on the probability of a patient being alive from non-cardiovascular causes during each time interval, using a similar method as for the inverse probability of censoring weight, as

$$D_{t}=\prod_{k=0}^{t} \frac{1}{Pr(D_{k}=0|D_{k-1}=0,Y_{k-1}=0,\bar{A},V)}$$

where Y=outcome, D=death, A=treatment assignment, V=baseline characteristics, t=time interval since baseline

The final weight is the product between the inverse probability of censoring weight and death weight.

This approach allowed us to estimate the controlled direct effect of treatment strategies on cardiovascular outcomes, under a hypothetical scenario where no competing risk of deaths occurred ^2^. While appropriate for our etiological aim of quantifying the cardiotoxicity of fluoropyrimidine treatment through its direct pharmacological effect ^3^, this method overestimates cumulative incidence compared to real-world observations, as it assumes no competing risk event can occur.

Another approach for handling competing risk event is subdistributional hazard models. In contrast, the subdistributional hazard models estimate the total treatment effect, including pathways mediated by competing risk event, providing more accurate cumulative incidence functions. However, they are less suitable for our causal question of interest without insolating the anticipated survival benefits of fluoropyrimidines; that is, fluoropyrimidine treatment would appear to increase the probability of cardiovascular events by reducing the risk of mortality. Results from the subdistributional hazard models would not have clear causal interpretations ^3^.

Thus, the use of a cause-specific hazard model with weighting was deliberately chosen to align with our etiological aim, acknowledging the trade-off in cumulative incidence estimation. In a sensitivity analysis, we used a different approach to address the competing risk event by investigating the treatment effect on the composite outcome of death and cardiovascular events.

# Supplemental Methods 3. Rationales for the subgroup and sensitivity analysis.

1. In the subgroup analysis, we stratified the cohorts by certain important confounding variables to further reduce the heterogeneity between patient groups and residual confounding, as well as to explore any effect modification by the baseline factors ^4^.
2. We conducted additional analysis on the composite outcome of all-cause mortality and cardiovascular events. In this analysis, we eliminated competing risk events by treating it as part of the outcome. Results from this analysis can be interpreted as cardiovascular events-free survival, which can be used to directly compare the benefit in survival with the risk in cardiovascular events.
3. In the analysis of adjusting for the performance status (PS), we conducted a complete case analysis by excluding patients with missing baseline PS due to the substantial missingness. We also excluded patients with PS of 4 because these patients are usually considered ineligible for chemotherapy ^5,6^. We then included PS as an additional regressor in weight calculations and outcome models as PS might be an important confounder for chemotherapy and cardiovascular disease or mortality outcomes.
4. We extended the grace period from 8 weeks to 12 and 16 weeks. This is to capture more patients receiving fluoropyrimidine as adjuvant chemotherapy after surgery in the fluoropyrimidine group.
5. We truncated the weight at 99.5^th^ percentile rather than 99^th^, as the truncation is a trade-off between precision and accuracy of the weighted analysis, and the optimal threshold of weight truncation is unclear ^7^.
6. We further added a cubic term of the time into the model to allow more flexibility of the risk prediction over time. In this analysis, we added time in its linear, quadratic, and cubic terms, and their interaction terms with the treatment indicator to predict the risk of outcomes at each time.
7. We analyzed non-specific chest pain coded in HES (ICD-10 codes R07.3 and R07.4) as an additional outcome as non-specific chest pain was reported as the most common fluoropyrimidine-associated cardiotoxicity ^8^.
8. We conducted a negative control outcome analysis with age-related cataract as the negative control outcome. We excluded patients with cataract at baseline. This negative control outcome analysis was used to quantitatively evaluate the residual confounding in the analysis. In our study, the residual confounding may be due to the lack of linkage to the primary care records, e.g., information on smoking status and body mass index. Age-related cataract was selected given its known association with obesity ^9^ and smoking ^10^, but not fluoropyrimidine treatment. So, we do not expect to see an association between fluoropyrimidine treatment and the risk of age-related cataract.
9. We explored the one-year event rate for each study endpoint in patients with baseline CAD as pre-existing CAD was suggested as a pre-disposing factor for fluoropyrimidine-associated cardiotoxicity ^11^.
10. Lastly, we explored long-term effect of fluoropyrimidine treatment on outcomes up to five years following the cancer diagnosis. As NICOR data was only available until 2019, we did not use the linkages to NICOR to ascertain cardiovascular events in this analysis.

# Supplemental Table 3. Inverse probability weight distribution for death outcome.

|  | **Before truncation** | | **After truncation** | |
| --- | --- | --- | --- | --- |
| **Measurement** | **Fluoropyrimidine** | **No fluoropyrimidine** | **Fluoropyrimidine** | **No fluoropyrimidine** |
| 1^st^ percentile | 1.00 | 1.01 | 1.00 | 1.01 |
| 25^th^ percentile | 1.00 | 1.12 | 1.00 | 1.12 |
| 50^th^ percentile | 1.26 | 1.38 | 1.26 | 1.38 |
| 75^th^ percentile | 3.03 | 1.97 | 3.03 | 1.97 |
| 90^th^ percentile | 7.61 | 3.21 | 7.61 | 3.21 |
| 95^th^ percentile | 14.82 | 4.94 | 14.82 | 4.94 |
| 99^th^ percentile | 73.84 | 14.60 | 40.76 | 14.60 |
| Max | 2,863.82 | 23300.36 | 40.76 | 40.76 |
| Mean | 5.89 | 3.01 | 3.70 | 2.12 |
| SD | 39.14 | 99.91 | 6.75 | 3.15 |

SD, standard deviation.

# Supplemental Table 4. Inverse probability weight distribution for composite cardiovascular events outcome.

|  | **Before truncation** | | **After truncation** | |
| --- | --- | --- | --- | --- |
| **Measurement** | **Fluoropyrimidine** | **No fluoropyrimidine** | **Fluoropyrimidine** | **No fluoropyrimidine** |
| 1^st^ percentile | 1.00 | 1.02 | 1.00 | 1.02 |
| 25^th^ percentile | 1.04 | 1.21 | 1.04 | 1.21 |
| 50^th^ percentile | 1.55 | 1.69 | 1.55 | 1.69 |
| 75^th^ percentile | 3.89 | 2.73 | 3.89 | 2.73 |
| 90^th^ percentile | 10.10 | 5.02 | 10.10 | 5.02 |
| 95^th^ percentile | 20.39 | 8.04 | 20.39 | 8.04 |
| 99^th^ percentile | 113.23 | 26.90 | 62.55 | 26.90 |
| Max | 16,917.57 | 39,827.58 | 62.55 | 62.55 |
| Mean | 8.45 | 4.39 | 4.98 | 3.01 |
| SD | 69.69 | 116.10 | 10.15 | 5.38 |

SD, standard deviation.


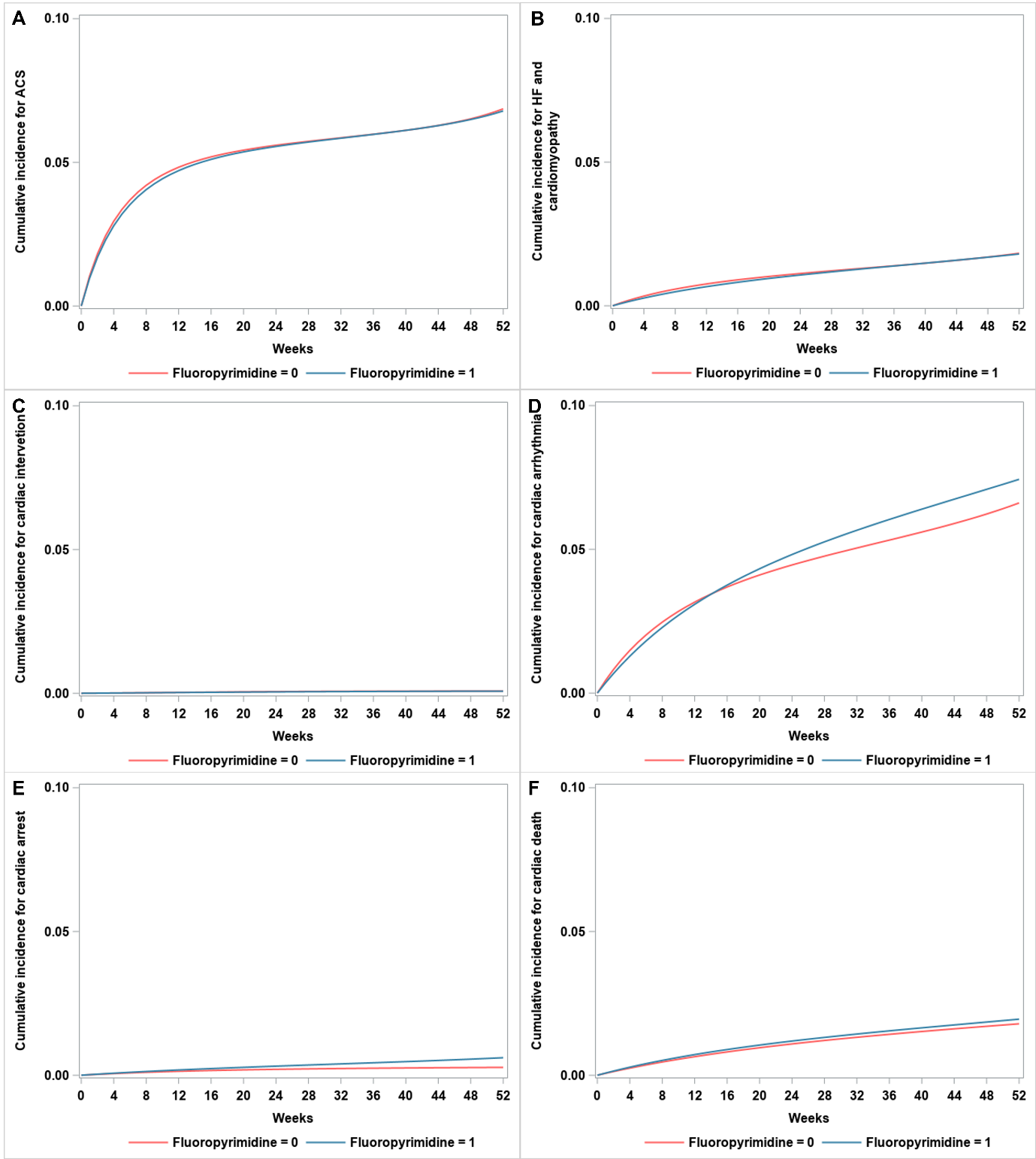


# Supplemental Figure 1. Weighted, standardized cumulative incidence curves for (A) acute coronary syndrome; (B) heart failure and cardiomyopathy; (C) cardiac intervention; (D) cardiac arrhythmia; (E) cardiac arrest; (F) cardiac death comparing patients receiving fluoropyrimidine versus no fluoropyrimidine treatment.

# Supplemental Table 5. One-year risk differences, risk ratios, and hazard ratios for individual cardiovascular events comparing patients on fluoropyrimidine versus no fluoropyrimidine.

| **Treatment** | **No. of patients** | **No. of patient-years** | **No. of outcomes** | **1-year risk difference (%)**  **(95% CI)** | **One-year risk ratio**  **(95% CI)** | **Hazard ratio**  **(95% CI)** |  |
| --- | --- | --- | --- | --- | --- | --- | --- |
| **ACS** | | | | | | |  |
| Fluoropyrimidine | 103,110 | 31,281 | 4,244 | -0.07  (-0.63 to 0.51) | 0.99  (0.91 to 1.08) | 0.99  (0.90 to 1.08) |  |
|  |  |  |  |  |  |  |  |
| No fluoropyrimidine | 103,110 | 34,228 | 4,908 | Reference | Reference | Reference |  |
|  |  |  |  |  |  |  |  |
| **HF and cardiomyopathy** | | | | | | |  |
| Fluoropyrimidine | 103,110 | 31,281 | 614 | -0.03  (-0.49 to 0.42) | 0.99  (0.76 to 1.23) | 0.98  (0.77 to 1.25) |  |
|  |  |  |  |  |  |  |  |
| No fluoropyrimidine | 103,110 | 34,228 | 907 | Reference | Reference | Reference |  |
|  |  |  |  |  |  |  |  |
| **Cardiac intervention** | | | | | | |  |
| Fluoropyrimidine | 103,110 | 31,281 | 24 | 0.00  (-0.06 to 0.06) | 0.99  (0.38 to 1.92) | 0.97  (0.39 to 2.42) |  |
|  |  |  |  |  |  |  |  |
| No fluoropyrimidine | 103,110 | 34,228 | 34 | Reference | Reference | Reference |  |
|  |  |  |  |  |  |  |  |
| **Cardiac arrhythmia** | | | | | | |  |
| Fluoropyrimidine | 103,110 | 31,281 | 2,979 | 0.82  (0.11 to 1.58) | 1.12  (1.02 to 1.25) | 1.14  (1.01 to 1.29) |  |
|  |  |  |  |  |  |  |  |
| No fluoropyrimidine | 103,110 | 34,228 | 3,654 | Reference | Reference | Reference |  |
|  |  |  |  |  |  |  |  |
| **Cardiac arrest** | | | | | | |  |
| Fluoropyrimidine | 103,110 | 31,281 | 179 | 0.34  (0.13 to 0.47) | 2.23  (1.39 to 2.78) | 2.24  (1.53 to 3.27) |  |
|  |  |  |  |  |  |  |  |
| No fluoropyrimidine | 103,110 | 34,228 | 160 | Reference | Reference | Reference |  |
|  |  |  |  |  |  |  |  |
| **Cardiac death** | | | | | | |  |
| Fluoropyrimidine | 103,110 | 31,281 | 620 | 0.16  (-0.22 to 0.47) | 1.09  (0.89 to 1.27) | 1.09  (0.87 to 1.36) |  |
|  |  |  |  |  |  |  |  |
| No fluoropyrimidine | 103,110 | 34,228 | 748 | Reference | Reference | Reference |  |
|  |  |  |  |  |  |  |  |

# Supplemental Table 6. One-year absolute risks, risk differences, risk ratios, and hazard ratios for all-cause mortality comparing patients on fluoropyrimidine versus no fluoropyrimidine, stratified by subgroups.

| **Subgroup** | **Treatment with fluoropyrimidine** | **Number of patients** | **1-year absolute risk (%)**  **(95% CI)** | **1-year risk difference (%)**  **(95% CI)** | **1-year risk ratio**  **(95% CI)** | **Hazard ratio**  **(95% CI)** |
| --- | --- | --- | --- | --- | --- | --- |
| Age >= 60 years | Yes | 81,889 | 44.2 (43.4 to 45.2) | -8.5 (-9.4 to -7.3) | 0.84 (0.82 to 0.86) | 0.69 (0.67 to 0.72) |
|  | No | 81,889 | 52.7 (52.0 to 53.3) | Reference | Reference | Reference |
| Age < 60 years | Yes | 21,221 | 33.2 (31.7 to 34.6) | -3.6 (-5.4 to -1.4) | 0.90 (0.86 to 0.96) | 0.80 (0.73 to 0.90) |
|  | No | 21,221 | 36.8 (35.1 to 38.2) | Reference | Reference | Reference |
| Male | Yes | 61,517 | 41.9 (41.0 to 42.8) | -8.5 (-9.6 to -7.2) | 0.83 (0.81 to 0.86) | 0.68 (0.65 to 0.71) |
|  | No | 61,517 | 50.4 (49.6 to 51.2) | Reference | Reference | Reference |
| Female | Yes | 41,593 | 41.9 (40.9 to 43.0) | -6.2 (-7.1 to -4.5) | 0.87 (0.85 to 0.90) | 0.75 (0.70 to 0.79) |
|  | No | 41,593 | 48.1 (47.1 to 48.8) | Reference | Reference | Reference |
| CVD | Yes | 24,554 | 52.7 (50.9 to 54.6) | -7.9 (-9.5 to -5.8) | 0.87 (0.84 to 0.90) | 0.72 (0.68 to 0.77) |
|  | No | 24,554 | 60.5 (59.4 to 61.3) | Reference | Reference | Reference |
| No CVD | Yes | 78,556 | 38.5 (37.7 to 39.3) | -7.6 (-8.7 to -6.4) | 0.83 (0.81 to 0.86) | 0.70 (0.67 to 0.73) |
|  | No | 78,556 | 46.1 (45.5 to 46.8) | Reference | Reference | Reference |
| Stage II to III | Yes | 55,900 | 20.2 (19.1 to 21.2) | -8.1 (-9.4 to -6.8) | 0.71 (0.67 to 0.75) | 0.63 (0.58 to 0.68) |
|  | No | 55,900 | 28.3 (27.5 to 29.1) | Reference | Reference | Reference |
| Stage IV | Yes | 47,210 | 66.4 (65.3 to 67.5) | -8.3 (-9.8 to -6.9) | 0.89 (0.87 to 0.91) | 0.72 (0.69 to 0.75) |
|  | No | 47,210 | 74.7 (73.8 to 75.5) | Reference | Reference | Reference |
| Esophageal cancer | Yes | 21,392 | 53.0 (51.1 to 55.0) | -15.8 (-17.8 to -13.7) | 0.77 (0.74 to 0.80) | 0.55 (0.52 to 0.60) |
|  | No | 21,392 | 68.8 (67.3 to 70.0) | Reference | Reference | Reference |
| Gastric cancer | Yes | 13,311 | 57.1 (55.3 to 58.9) | -14.1 (-16.2 to -11.6) | 0.80 (0.78 to 0.83) | 0.55 (0.51 to 0.60) |
|  | No | 13,311 | 71.1 (69.8 to 72.6) | Reference | Reference | Reference |
| Colorectal cancer | Yes | 68,407 | 35.1 (34.1 to 36.0) | -3.5 (-4.4 to -2.2) | 0.91 (0.89 to 0.94) | 0.83 (0.78 to 0.87) |
|  | No | 68,407 | 38.6 (37.9 to 39.1) | Reference | Reference | Reference |

# Supplemental Table 7. One-year absolute risks, risk differences, risk ratios, and hazard ratios for composite cardiovascular events comparing patients on fluoropyrimidine versus no fluoropyrimidine, stratified by subgroups.

| **Subgroup** | **Treatment with fluoropyrimidine** | **Number of patients** | **1-year absolute risk (%)**  **(95% CI)** | **1-year risk difference (%)**  **(95% CI)** | **1-year risk ratio**  **(95% CI)** | **Hazard ratio**  **(95% CI)** |
| --- | --- | --- | --- | --- | --- | --- |
| Age >= 60 years | Yes | 81,889 | 18.3 (17.4 to 19.3) | 0.6 (-0.3 to 1.6) | 1.03 (0.98 to 1.10) | 1.04 (0.96 to 1.12) |
|  | No | 81,889 | 17.7 (17.0 to 18.3) | Reference | Reference | Reference |
| Age < 60 years | Yes | 21,221 | 5.7 (5.0 to 6.4) | 0.2 (-0.7 to 1.3) | 1.03 (0.88 to 1.27) | 1.05 (0.81 to 1.34) |
|  | No | 21,221 | 5.5 (4.6 to 6.2) | Reference | Reference | Reference |
| Male | Yes | 61,517 | 18.0 (17.1 to 19.0) | 0.7 (-0.6 to 1.8) | 1.04 (0.97 to 1.11) | 1.05 (0.96 to 1.14) |
|  | No | 61,517 | 17.4 (16.6 to 18.0) | Reference | Reference | Reference |
| Female | Yes | 41,593 | 12.7 (11.7 to 14.2) | 1.4 (0.2 to 2.9) | 1.12 (1.02 to 1.26) | 1.14 (0.99 to 1.30) |
|  | No | 41,593 | 11.3 (10.7 to 12.0) | Reference | Reference | Reference |
| CVD | Yes | 24,554 | 41.1 (38.6 to 43.6) | 0.3 (-2.1 to 2.7) | 1.01 (0.95 to 1.07) | 0.99 (0.91 to 1.08) |
|  | No | 24,554 | 40.8 (39.2 to 41.8) | Reference | Reference | Reference |
| No CVD | Yes | 78,556 | 7.9 (7.2 to 8.5) | 0.7 (0.0 to 1.6) | 1.10 (1.00 to 1.23) | 1.12 (1.00 to 1.27) |
|  | No | 78,556 | 7.2 (6.7 to 7.7) | Reference | Reference | Reference |
| Stage II to III | Yes | 55,900 | 17.3 (16.5 to 18.4) | 1.5 (0.5 to 2.7) | 1.09 (1.03 to 1.18) | 1.10 (1.02 to 1.20) |
|  | No | 55,900 | 15.9 (15.3 to 16.4) | Reference | Reference | Reference |
| Stage IV | Yes | 47,210 | 13.6 (12.6 to 14.8) | -0.1 (-1.4 to 1.3) | 1.00 (0.91 to 1.11) | 1.01 (0.88 to 1.15) |
|  | No | 47,210 | 13.7 (12.8 to 14.4) | Reference | Reference | Reference |
| Esophageal cancer | Yes | 21,392 | 22.3 (20.4 to 24.2) | 2.5 (0.5 to 4.6) | 1.13 (1.02 to 1.25) | 1.14 (0.99 to 1.30) |
|  | No | 21,392 | 19.8 (18.3 to 21.0) | Reference | Reference | Reference |
| Gastric cancer | Yes | 13,311 | 21.5 (18.9 to 23.5) | 4.6 (1.4 to 7.2) | 1.27 (1.08 to 1.45) | 1.33 (1.11 to 1.61) |
|  | No | 13,311 | 16.9 (15.2 to 18.2) | Reference | Reference | Reference |
| Colorectal cancer | Yes | 68,407 | 12.6 (11.8 to 13.5) | -0.3 (-1.2 to 0.7) | 0.97 (0.91 to 1.05) | 0.97 (0.89 to 1.06) |
|  | No | 68,407 | 13.0 (12.4 to 13.4) | Reference | Reference | Reference |

# Supplemental Table 8. Sensitivity analysis: composite outcome of all-cause mortality and cardiovascular events and the point estimates for one-year absolute risk, risk difference, risk ratio, and hazard ratio.

| **Treatment** | **No. of patients** | **No. of patient-years** | **No. of outcomes** | **1-year absolute risk (%)** | **1-year risk difference (%)** | **1-year risk ratio** | **Hazard ratio** |  |
| --- | --- | --- | --- | --- | --- | --- | --- | --- |
| Fluoropyrimidine | 103,110 | 31,281 | 27,378 | 49.4 | -6.4 | 0.89 | 0.79 |  |
|  |  |  |  |  |  |  |  |  |
| No fluoropyrimidine | 103,110 | 34,228 | 37,152 | 55.8 | Reference | Reference | Reference |  |
|  |  |  |  |  |  |  |  |  |

# Supplemental Table 9. **Distribution of performance status of the study cohorts, before and after the grace period.**

|  | **All patients (n=103,110)** | **Fluoropyrimidine - after grace period (n=25,401)** | **No fluoropyrimidine - after grace period (n=64,589)** |
| --- | --- | --- | --- |
| **Performance status, n (%)** |  |  |  |
| 0 | 28,150 (27.3) | 9,603 (39.2) | 17,761 (27.1) |
| 1 | 19,349 (18.8) | 5,466 (22.3) | 12,671 (19.3) |
| 2 | 8,485 (8.2) | 1,180 (4.8) | 5,916 (9.0) |
| 3 | 4,899 (4.8) | 191 (0.8) | 2,977 (4.5) |
| 4 | 965 (0.9) | 19 (0.1) | 380 (0.6) |
| Unknown | 41,262 (40.0) | 8,028 (32.8) | 25,798 (39.4) |

# Supplemental Table 10. Sensitivity analysis: performance status being controlled and the point estimates for one-year absolute risk, risk difference, risk ratio, and hazard ratio for all-cause mortality and composite cardiovascular events.

| **Treatment** | **No. of patients** | **No. of patient-years** | **No. of outcomes** | **1-year absolute risk (%)** | **1-year risk difference (%)** | **1-year risk ratio** | **Hazard ratio** |  |
| --- | --- | --- | --- | --- | --- | --- | --- | --- |
| **All-cause mortality** | | | | | | | |  |
| Fluoropyrimidine | 60,883 | 21,446 | 10,275 | 38.6 | -6.2 | 0.86 | 0.74 |  |
|  |  |  |  |  |  |  |  |  |
| No fluoropyrimidine | 60,883 | 22,169 | 15,024 | 44.8 | Reference | Reference | Reference |  |
|  |  |  |  |  |  |  |  |  |
| **Composite cardiovascular events** | | | | | | | |  |
| Fluoropyrimidine | 60,883 | 20,135 | 5,149 | 16.1 | 1.2 | 1.08 | 1.10 |  |
|  |  |  |  |  |  |  |  |  |
| No fluoropyrimidine | 60,883 | 20,070 | 5,945 | 14.9 | Reference | Reference | Reference |  |
|  |  |  |  |  |  |  |  |  |

# Supplemental Table 11. Sensitivity analysis: 12-week grace period and the point estimates for one-year absolute risk, risk difference, risk ratio, and hazard ratio for all-cause mortality and composite cardiovascular events.

| **Treatment** | **No. of patients** | **No. of patient-years** | **No. of outcomes** | **1-year absolute risk (%)** | **1-year risk difference (%)** | **1-year risk ratio** | **Hazard ratio** |  |
| --- | --- | --- | --- | --- | --- | --- | --- | --- |
| **All-cause mortality** | | | | | | | |  |
| Fluoropyrimidine | 103,110 | 38,021 | 25,172 | 42.3 | -7.2 | 0.86 | 0.72 |  |
|  |  |  |  |  |  |  |  |  |
| No fluoropyrimidine | 103,110 | 37,657 | 31,104 | 49.5 | Reference | Reference | Reference |  |
|  |  |  |  |  |  |  |  |  |
| **Composite cardiovascular events** | | | | | | | |  |
| Fluoropyrimidine | 103,110 | 35,748 | 9,276 | 16.2 | 1.0 | 1.06 | 1.09 |  |
|  |  |  |  |  |  |  |  |  |
| No fluoropyrimidine | 103,110 | 34,228 | 9,873 | 15.2 | Reference | Reference | Reference |  |
|  |  |  |  |  |  |  |  |  |

# Supplemental Table 12. Sensitivity analysis: 16-week grace period and the point estimates for one-year absolute risk, risk difference, risk ratio, and hazard ratio for all-cause mortality and composite cardiovascular events.

| **Treatment** | **No. of patients** | **No. of patient-years** | **No. of outcomes** | **1-year absolute risk (%)** | **1-year risk difference (%)** | **1-year risk ratio** | **Hazard ratio** |  |
| --- | --- | --- | --- | --- | --- | --- | --- | --- |
| **All-cause mortality** | | | | | | | |  |
| Fluoropyrimidine | 103,110 | 42,582 | 28,318 | 42.2 | -7.1 | 0.86 | 0.73 |  |
|  |  |  |  |  |  |  |  |  |
| No fluoropyrimidine | 103,110 | 37,657 | 31,104 | 49.3 | Reference | Reference | Reference |  |
|  |  |  |  |  |  |  |  |  |
| **Composite cardiovascular events** | | | | | | | |  |
| Fluoropyrimidine | 103,110 | 39,899 | 10,018 | 16.5 | 1.1 | 1.08 | 1.10 |  |
|  |  |  |  |  |  |  |  |  |
| No fluoropyrimidine | 103,110 | 34,228 | 9,873 | 15.3 | Reference | Reference | Reference |  |
|  |  |  |  |  |  |  |  |  |

# Supplemental Table 13. Sensitivity analysis: weight truncation at 99.5^th^ percentile and the point estimates for one-year absolute risk, risk difference, risk ratio, and hazard ratio for all-cause mortality and composite cardiovascular events.

| **Treatment** | **No. of patients** | **No. of patient-years** | **No. of outcomes** | **1-year absolute risk (%)** | **1-year risk difference (%)** | **1-year risk ratio** | **Hazard ratio** |  |
| --- | --- | --- | --- | --- | --- | --- | --- | --- |
| **All-cause mortality** | | | | | | | |  |
| Fluoropyrimidine | 103,110 | 33,178 | 21,110 | 42.2 | -7.5 | 0.84 | 0.71 |  |
|  |  |  |  |  |  |  |  |  |
| No fluoropyrimidine | 103,110 | 37,657 | 31,104 | 49.8 | Reference | Reference | Reference |  |
|  |  |  |  |  |  |  |  |  |
| **Composite cardiovascular events** | | | | | | | |  |
| Fluoropyrimidine | 103,110 | 31,281 | 8,287 | 15.6 | 0.8 | 1.06 | 1.08 |  |
|  |  |  |  |  |  |  |  |  |
| No fluoropyrimidine | 103,110 | 34,228 | 9,873 | 14.8 | Reference | Reference | Reference |  |
|  |  |  |  |  |  |  |  |  |

# Supplemental Table 14. Sensitivity analysis: time added in its linear, quadratic, and cubic terms and the point estimates for one-year absolute risk, risk difference, risk ratio, and hazard ratio for all-cause mortality and composite cardiovascular events.

| **Treatment** | **No. of patients** | **No. of patient-years** | **No. of outcomes** | **1-year absolute risk (%)** | **1-year risk difference (%)** | **1-year risk ratio** | **Hazard ratio** |  |
| --- | --- | --- | --- | --- | --- | --- | --- | --- |
| **All-cause mortality** | | | | | | | |  |
| Fluoropyrimidine | 103,110 | 33,178 | 21,110 | 41.9 | -7.7 | 0.84 | 0.70 |  |
|  |  |  |  |  |  |  |  |  |
| No fluoropyrimidine | 103,110 | 37,657 | 31,104 | 49.6 | Reference | Reference | Reference |  |
|  |  |  |  |  |  |  |  |  |
| **Composite cardiovascular events** | | | | | | | |  |
| Fluoropyrimidine | 103,110 | 31,281 | 8,287 | 15.9 | 1.0 | 1.07 | 1.09 |  |
|  |  |  |  |  |  |  |  |  |
| No fluoropyrimidine | 103,110 | 34,228 | 9,873 | 14.9 | Reference | Reference | Reference |  |
|  |  |  |  |  |  |  |  |  |

# Supplemental Table 15. Sensitivity analysis: one-year risk differences, risk ratios, and hazard ratios for non-specific chest pain comparing patients on fluoropyrimidine versus no fluoropyrimidine.

| **Treatment** | **No. of patients** | **No. of patient-years** | **No. of outcomes** | **1-year risk difference (%)**  **(95% CI)** | **1-year risk ratio**  **(95% CI)** | **Hazard ratio**  **(95% CI)** |  |
| --- | --- | --- | --- | --- | --- | --- | --- |
| Fluoropyrimidine | 103,110 | 30,972 | 1,045 | 0.57  (-0.01 to 1.14) | 1.22  (1.00 to 1.48) | 1.19  (0.98 to 1.45) |  |
|  |  |  |  |  |  |  |  |
| No fluoropyrimidine | 103,110 | 34,088 | 927 | Reference | Reference | Reference |  |
|  |  |  |  |  |  |  |  |

# Supplemental Table 16. Exploratory analysis: one-year absolute risk, risk difference, and risk ratio of each study outcome comparing patients on fluoropyrimidine versus no fluoropyrimidine, among patients with baseline coronary artery disease.

| **Outcome** | **Treatment** | **1-year absolute risk (%)** | **1-year risk difference (%)** | **1-year risk ratio** |
| --- | --- | --- | --- | --- |
| Death | Fluoropyrimidine | 49.4 | -8.6 | 0.85 |
|  | No fluoropyrimidine | 58.0 | Reference | Reference |
| Composite cardiovascular events | Fluoropyrimidine | 52.5 | 0.0 | 1.00 |
|  | No fluoropyrimidine | 52.5 | Reference | Reference |
| ACS | Fluoropyrimidine | 42.0 | 0.7 | 1.02 |
|  | No fluoropyrimidine | 41.3 | Reference | Reference |
| HF and cardiomyopathy | Fluoropyrimidine | 5.7 | -0.2 | 0.97 |
|  | No fluoropyrimidine | 5.9 | Reference | Reference |
| Arrhythmia | Fluoropyrimidine | 13.1 | -0.2 | 0.98 |
|  | No fluoropyrimidine | 13.3 | Reference | Reference |
| Cardiac death | Fluoropyrimidine | 3.3 | 0.4 | 1.14 |
|  | No fluoropyrimidine | 2.9 | Reference | Reference |
| Cardiac arrest | Fluoropyrimidine | 0.5 | -0.1 | 0.83 |
|  | No fluoropyrimidine | 0.6 | Reference | Reference |

# Supplemental Table 17. Exploratory analysis: five-year absolute risks, risk differences, risk ratios, and hazard ratios for all-cause mortality and composite cardiovascular events comparing patients on fluoropyrimidine and versus no fluoropyrimidine.

| **Treatment** | **No. of patients** | **No. of patient-years** | **No. of outcomes** | **5-year absolute risk (%)** | **5-year risk difference (%)** | **5-year risk ratio** | **Hazard ratio**  **(95% CI)** |  |
| --- | --- | --- | --- | --- | --- | --- | --- | --- |
| **All-cause mortality** | | | | | | | |  |
| Fluoropyrimidine | 103,110 | 62,849 | 31,134 | 70.5 | 10.3 | 0.87 | 0.73  (0.71 to 0.74) |  |
|  |  |  |  |  |  |  |  |  |
| No fluoropyrimidine | 103,110 | 72,935 | 40,547 | 80.8 | Reference | Reference | Reference |  |
|  |  |  |  |  |  |  |  |  |
| **Composite cardiovascular events** | | | | | | | |  |
| Fluoropyrimidine | 103,110 | 57,142 | 8,385 | 27.9 | -2.0 | 0.93 | 0.95  (0.90 to 1.00) |  |
|  |  |  |  |  |  |  |  |  |
| No fluoropyrimidine | 103,110 | 62,069 | 10,486 | 29.9 | Reference | Reference | Reference |  |
|  |  |  |  |  |  |  |  |  |

CI, confidence interval.

**
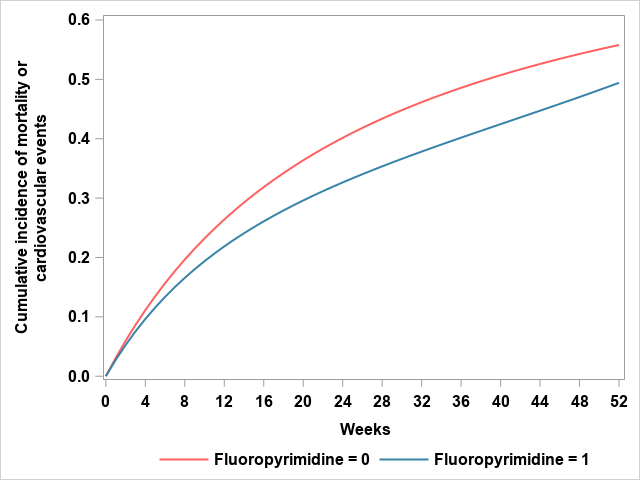
**

# Supplemental Figure 2. Weighted, standardized cumulative incidence curves for composite all-cause mortality and cardiovascular events comparing patients on fluoropyrimidine versus no fluoropyrimidine.


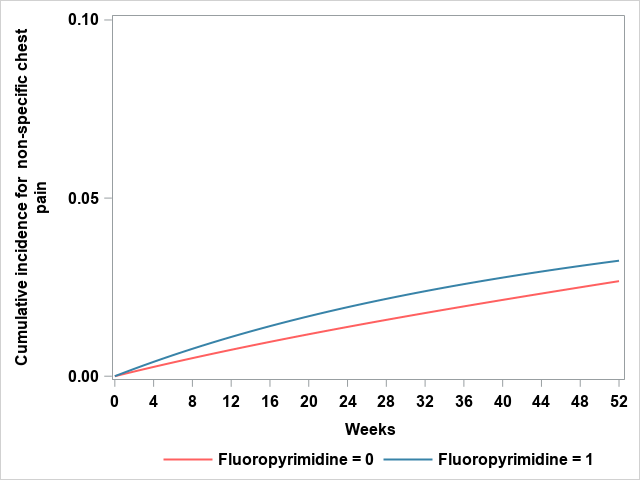


# Supplemental Figure 3. Weighted, standardized cumulative incidence curves for non-specific chest pain comparing patients on fluoropyrimidine versus no fluoropyrimidine.

# References

1. Henson KE, Elliss-Brookes L, Coupland VH, et al. Data Resource Profile: National Cancer Registration Dataset in England. *Int J Epidemiol*. 2020;49:16–16h.

2. Young JG, Stensrud MJ, Tchetgen Tchetgen EJ, Hernán MA. A causal framework for classical statistical estimands in failure-time settings with competing events. *Stat Med*. 2020;39:1199–1236.

3. Mansournia MA, Nazemipour M, Etminan M. A practical guide to handling competing events in etiologic time-to-event studies. *Glob Epidemiol*. 2022;4:100080.

4. Anon. Causal Inference: What If (the book) | Miguel Hernan’s Faculty Website | Harvard T.H. Chan School of Public Health Accessed December 7, 2023. https://www.hsph.harvard.edu/miguel-hernan/causal-inference-book/.

5. Scott JM, Stene G, Edvardsen E, Jones LW. Performance Status in Cancer: Not Broken, But Time for an Upgrade? *J Clin Oncol*. 2020;38:2824–2829.

6. Denduluri N, Patt DA, Wang Y, et al. Dose Delays, Dose Reductions, and Relative Dose Intensity in Patients With Cancer Who Received Adjuvant or Neoadjuvant Chemotherapy in Community Oncology Practices. *Journal of the National Comprehensive Cancer Network*. 2015;13:1383–1393.

7. Xiao Y, Moodie EEM, Abrahamowicz M. Comparison of approaches to weight truncation for marginal structural Cox models. *Epidemiol Methods*. 2013;2:1–20.

8. Padegimas A, Carver JR. How to Diagnose and Manage Patients With Fluoropyrimidine-Induced Chest Pain: A Single Center Approach. *JACC CardioOncol*. 2020;2:650–654.

9. Ye J, Lou LX, He JJ, Xu YF. Body Mass Index and Risk of Age-Related Cataract: A Meta-Analysis of Prospective Cohort Studies. *PLoS One*. 2014;9.

10. Raju P, George R, Ve Ramesh S, Arvind H, Baskaran M, Vijaya L. Influence of tobacco use on cataract development. *British Journal of Ophthalmology*. 2006;90:1374–1377.

11. Zamorano JL, Lancellotti P, Rodriguez Muñoz D, et al. 2016 ESC Position Paper on cancer treatments and cardiovascular toxicity developed under the auspices of the ESC Committee for Practice Guidelines. *Eur Heart J*. 2016.
